# Supplementary material for: TLR3 forms a highly organized cluster when bound to a poly(I:C) RNA ligand
Source: Nat Commun. 2022 Nov 12;13:6876. doi: 10.1038/s41467-022-34602-0 (PMC9653405; doi:10.1038/s41467-022-34602-0)
Supplement: Supplementary file 1 — Supplementary Information [file 41467_2022_34602_MOESM1_ESM.pdf]

SUPPLEMENTARY INFORMATION

TLR3 forms a highly organized cluster when bound to a poly(I:C) RNA ligand

Lim et al.

Supplementary Table 1 | Cryo-EM data collection, refinement and validation statistics

	TLR3-poly(I:C) clustered (EMD-32844) (PDB 7WV3)		
	**Consensus**	**dimer 1 focus**	**dimer 2 focus**
**Sample condition**			
TLR concentration (mg/ml)	7.2	7.2	7.2
poly (I:C) concentration (mg/ml)	0.41	0.41	0.41
TLR3/available binding site ratio	1.8	1.8	1.8
**Data collection and processing**			
Magnification	100,500 x	100,500 x	100,500 x
Voltage (kV)	300	300	300
Electron exposure (e^-/Å^2)	50	50	50
Defocus range (μm)	-0.7 to -2.1	-0.7 to -2.1	-0.7 to -2.1
Pixel size (Å)	0.85	0.85	0.85
Box size (pixels)	480	480	480
Symmetry imposed	C1	C1	C1
Initial particle images (no.)	1,435,549	1,435,549	1,435,549
Final particle images (no.)	892,514	892,514	892,514
Map resolution (Å)	2.26	2.26	2.32
FSC threshold	0.143	0.143	0.143
**Refinement**			
Initial model used (PDB code)	2A0Z, 3CIY		
Model resolution (Å)	2.8		
FSC threshold	0.5		
Model composition			
Non-hydrogen atoms	25,386		
Protein residues	2,672		
Nucleotide residues	160		
Glycan residues (NAG)	44		
B factors (Å^2)			
Protein	49.31		
Glycan residues	51.34		
Nucleotides	105.19		
R.m.s. deviations			
Bond lengths (Å)	0.007		
Bond angles (°)	0.601		
Validation			
MolProbity score	1.46		
Clashscore	4.46		
Poor rotamers (%)	0.32		
Ramanchandran plot			
Favored (%)	96.40		
Allowed (%)	3.60		
Disallowed (%)	0.00		

Supplementary Table 2 | Cryo-EM data collection, refinement and validation statistics

	ectoTLR3-poly(I:C) clustered (EMD-32845) (PDB 7WV4)		
	**Consensus**	**dimer 1 focus**	**dimer 2 focus**
**Sample condition**			
TLR concentration (mg/ml)	1.7	1.7	1.7
poly (I:C) concentration (mg/ml)	0.50	0.50	0.50
TLR3/available binding site ratio	0.47	0.47	0.47
**Data collection and processing**			
Magnification	100,000x	100,000x	100,000x
Voltage (kV)	200	200	200
Electron exposure (e^-/Å^2)	50	50	50
Defocus range (μm)	-1.3 to -2.5	-1.3 to -2.5	-1.3 to -2.5
Pixel size (Å)	0.831	0.831	0.831
Box size (pixels)	512	512	512
Symmetry imposed	C1	C1	C1
Initial particle images (no.)	337,258	337,258	337,258
Final particle images (no.)	59,774	59,774	59,774
Map resolution (Å)	3.35	3.35	3.49
FSC threshold	0.143	0.143	0.143
**Refinement**			
Initial model used (PDB code)	2A0Z, 3CIY		
Model resolution (Å)	4.2		
FSC threshold	0.5		
Model composition			
Non-hydrogen atoms	24,490		
Protein residues	2,636		
Nucleotide residues	160		
B factors (Å^2)			
Protein	120.77		
Nucleotides	153.73		
R.m.s. deviations			
Bond lengths (Å)	0.002		
Bond angles (°)	0.595		
Validation			
MolProbity score	1.73		
Clashscore	7.60		
Poor rotamers (%)	0.00		
Ramanchandran plot			
Favored (%)	95.43		
Allowed (%)	4.57		
Disallowed (%)	0.00		

Supplementary Table 3 | Cryo-EM data collection, refinement and validation statistics

	ectoTLR3-poly(I:C) (EMD-32846) (PDB 7WV5)		
	**ratio 0.47**	**ratio 0.34**	**ratio 0.17**
**Sample condition**			
TLR concentration (mg/ml)	1.7	1.0	0.5
poly I:C concentration (mg/ml)	0.50	0.41	0.41
TLR3/available binding site ratio	0.47	0.34	0.17
**Data collection and processing**			
Magnification	100,000x	100,000x	100,000x
Voltage (kV)	200	200	200
Electron exposure (e^-/Å^2)	50	50	50
Defocus range (μm)	-1.3 to -2.5	-0.9 to -2.1	-0.7 to -2.1
Pixel size (Å)	0.831	0.831	0.831
Box size (pixels)	480		
Symmetry imposed	C2		
Initial particle images (no.)	337,258		
Final particle images (no.)	84,157		
Map resolution (Å)	3.1		
FSC threshold	0.143		
**Refinement**			
Initial model used (PDB code)	2A0Z, 3CIY		
Model resolution (Å)	3.6		
FSC threshold	0.5		
Model composition			
Non-hydrogen atoms	12,958		
Protein residues	1,318		
Nucleotide residues	92		
Glycan residues (NAG, BMA)	30, 4		
B factors (Å^2)			
Protein	82.02		
Glycan residues	96.66		
Nucleotides	130.54		
R.m.s. deviations			
Bond lengths (Å)	0.005		
Bond angles (°)	0.496		
Validation			
MolProbity score	1.49		
Clashscore	5.50		
Poor rotamers (%)	0.16		
Ramanchandran plot			
Favored (%)	96.80		
Allowed (%)	3.20		
Disallowed (%)	0.00		

Supplementary Table 4 | Cryo-EM data collection, refinement and validation statistics

	ectoTLR3-mAb12-poly(I:C)	ectoTLR-poly(I:C)	
		NT-mut	CT-mut
	(EMD-32852) (PDB 7WVF)	(EMD-32853) (PDB 7WVJ)	(EMD-32851) (PDB 7WVE)
**Sample condition**			
TLR concentration (mg/ml)	1.0	1.0	1.0
poly (I:C) concentration (mg/ml)	0.41	0.41	0.41
TLR3/available binding site ratio	0.34	0.34	0.34
**Data collection and processing**			
Magnification	92,000x	100,000x	100,000x
Voltage (kV)	200	200	200
Electron exposure (e^-/Å^2)	40	40	40
Defocus range (μm)	-0.9 to -2.5	-0.9 to -2.5	-0.9 to -2.5
Pixel size (Å)	1.113	0.831	0.831
Box size (pixels)	360	360	360
Symmetry imposed	C1	C2	C2
Initial particle images (no.)	340,110	152,957	156,678
Final particle images (no.)	136,838	39,645	86,284
Map resolution (Å)	3.91	3.26	3.11
FSC threshold	0.143	0.143	0.143
**Refinement**			
Initial model used (PDB code)	2A0Z, 3CIY, 3ULS	2A0Z, 3CIY	2A0Z, 3CIY
Model resolution (Å)	4.3	3.5	3.9
FSC threshold	0.5	0.5	0.5
Model composition			
Non-hydrogen atoms	14,249	12,498	12,488
Protein residues	1,546	1,318	1,318
Nucleotide residues	92	92	92
B factors (Å^2)			
Protein	118.46	77.75	71.31
Nucleotides	151.06	125.22	146.08
R.m.s. deviations			
Bond lengths (Å)	0.003	0.001	0.003
Bond angles (°)	0.509	0.389	0.601
Validation			
MolProbity score	1.58	1.45	1.74
Clashscore	6.62	4.98	6.46
Poor rotamers (%)	0.00	0.16	0.00
Ramanchandran plot			
Favored (%)	96.62	96.88	94.44
Allowed (%)	3.32	3.12	5.56
Disallowed (%)	0.07	0.00	0.00

Supplementary Table 5 | Cryo-EM data collection statistics

	TLR3-mAb12-poly(I:C)		TLR3(A795H)-poly(I:C)
	**Dimeric**	**Clustered**	**Clustered**
	**(EMD-34367)**		**(EMD-34361)**
**Sample condition**			
TLR concentration (mg/ml)	0.26	0.26	0.56
poly (I:C) concentration (mg/ml)	0.031	0.031	0.031
TLR3/available binding site ratio	0.86	0.86	1.85
**Data collection and processing**			
Magnification	100,000x	100,000x	100,500x
Voltage (kV)	200	200	300
Electron exposure ($e^-/Å^2$)	50	50	50
Defocus range (μm)	-1.0 to -2.4	-1.0 to -2.4	-0.7 to -2.4
Pixel size (Å)	0.831	0.831	0.850
Box size (pixels)	400	480	480
Symmetry imposed	C1	C1	C1
Initial particle images (no.)	6,891	1,005	45,216
Final particle images (no.)	6,864	976	37,215
Map resolution (Å)	13.2		6.47
FSC threshold	0.143		0.143

Supplementary Table 6 | TLR3 gene sequences for cryo-EM experiments

Protein name	Gene sequences
TLR3	CTCGAGCCACCATGAGACAGACTTTGCCTTGTATCTACTTTTGGGGGGGCCTTTTGCCCTTTGGGATGCTGTGTGCATCCTCCACC<br>ACCAAGTGCACTGTTAGCCATGAAGTTGCTGACTGCAGCCACCTGAAGTTGACTCAGGTACCCGATGATCTACCCACAAACATAAC<br>AGTGTTGAACCTTACCCATAATCAACTCAGAAGATTACCAGCCGCCAACTTCACAAGGTATAGCCAGCTAACTAGCTTGGATGTAGG<br>ATTTAACACCATCTCAAAACTGGAGCCAGAATTGTGCCAGAAACTTCCCATGTTAAAAGTTTTGAACCTCCAGCACAATGAGCTATCT<br>CAACTTTCTGATAAAACCTTTGCCTTCTGCACGAATTTGACTGAACTCCATCTCATGTCCAACTCAATCCAGAAAATTAAAAATAATCC<br>CTTTGTCAAGCAGAAGAATTTAATCACATTAGATCTGTCTCATAATGGCTTGTCATCTACAAAATTAGGAACTCAGGTTCAGCTGGAA<br>AATCTCCAAGAGCTTCTATTATCAAACAATAAAATTCAAGCGCTAAAAAGTGAAGAACTGGATATCTTTGCCAATTCATCTTTAAAAAA<br>ATTAGAGTTGTCATCGAATCAAATTAAAGAGTTTTCTCCAGGGTGTTTTCACGCAATTGGAAGATTATTTGGCCTCTTTCTGAACAAT<br>GTCCAGCTGGGTCCCAGCCTTACAGAGAAGCTATGTTTGGAATTAGCAAACACAAGCATTCGGAATCTGTCTCTGAGTAACAGCCA<br>GCTGTCCACCACCAGCAATACAACTTTCTTGGGACTAAAGTGGACAAATCTCACTATGCTCGATCTTTCCTACAACAACTTAAATGTG<br>GTTGGTAACGATTCCTTTGCTTGGCTTCCACAACTAGAATATTTCTTCCTAGAGTATAATAATATACAGCATTTGTTTTCTCACTCTTT<br>GCACGGGCTTTTCAATGTGAGGTACCTGAATTTGAAACGGTCTTTTACTAAACAAAGTATTTCCCTTGCCTCACTCCCCAAGATTGAT<br>GATTTTTCTTTTCAGTGGCTAAAATGTTTGGAGCACCTTAACATGGAAGATAATGATATTCCAGGCATAAAAAGCAATATGTTCACAG<br>GATTGATAAACCTGAAATACTTAAGTCTATCCAACTCCTTTACAAGTTTGCGAACTTTGACAAATGAAACATTTGTATCACTTGCTCAT<br>TCTCCCTTACACATACTCAACCTAACCAAGAATAAAATCTCAAAAATAGAGAGTGATGCTTTCTCTTGGTTGGGCCACCTAGAAGTAC<br>TTGACCTGGGCCTTAATGAAATTGGGCAAGAACTCACAGGCCAGGAATGGAGAGGTCTAGAAAATATTTTCGAAATCTATCTTTCCT<br>ACAACAAGTACCTGCAGCTGACTAGGAACTCCTTTGCCTTGGTCCCAAGCCTTCAACGACTGATGCTCCGAAGGGTGGCCCTTAAA<br>AATGTGGATAGCTCTCCTTCACCATTCCAGCCTCTTCGTAACTTGACCATTCTGGATCTAAGCAACAACAACATAGCCAACATAAATG<br>ATGACATGTTGGAGGGTCTTGAGAAACTAGAAATTCTCGATTTGCAGCATAACAACTTAGCACGGCTCTGGAAACACGCAAACCCTG<br>GTGGTCCCATTTATTTCCTAAAGGGTCTGTCTCACCTCCACATCCTTAACTTGGAGTCCAACGGCTTTGACGAGATCCCAGTTGAGG<br>TCTTCAAGGATTTATTTGAACTAAAGATCATCGATTTAGGATTGAATAATTTAAACACACTTCCAGCATCTGTCTTTAATAATCAGGTG<br>TCTCTAAAGTCATTGAACCTTCAGAAGAATCTCATAACATCCGTTGAGAAGAAGGTTTTCGGGCCAGCTTTCAGGAACCTGACTGAG<br>TTAGATATGCGCTTTAATCCCTTTGATTGCACGTGTGAAAGTATTGCCTGGTTTGTTAATTGGATTAACGAGACCCATACCAACATCC<br>CTGAGCTGTCAAGCCACTACCTTTGCAACACTCCACCTCACTATCATGGGTTCCCAGTGAGACTTTTTGATACATCATCTTGCAAAG<br>ACAGTGCCCCCTTTGAACTCTTTTTCATGATCAATACCAGTATCCTGTTGATTTTTATCTTTATTGTACTTCTCATCCACTTTGAGGGC<br>TGGAGGATATCTTTTTATTGGAATGTTTCAGTACATCGAGTTCTTGGTTTCAAAGAAATAGACAGACAGACAGAACAGTTTGAATATG<br>CAGCATATATAATTCATGCCTATAAAGATAAGGATTGGGTCTGGGAACATTTCTCTTCAATGGAAAAGGAAGACCAATCTCTCAAATT<br>TTGTCTGGAAGAAAGGGACTTTGAGGCGGGTGTTTTTGAACTAGAAGCAATTGTTAACAGCATCAAAAGAAGCAGAAAAATTATTTTT<br>GTTATAACACACCATCTATTAAAAGACCCATTATGCAAAAGATTCAAGGTACATCATGCAGTTCAACAAGCTATTGAACAAAATCTGG<br>ATTCCATTATATTGGTTTTCCTTGAGGAGATTCCAGATTATAAACTGAACCATGCACTCTGTTTGCGAAGAGGAATGTTTAAATCTCAC<br>TGCATCTTGAACTGGCCAGTTCAGAAAGAACGGATAGGTGCCTTTCGTCATAAATTGCAAGTAGCACTTGGATCCAAAAACTCTGTA<br>CATTCGAATTCTCTGGAGGTGCTGTTTCAGGGCCCTACCGCCGCCGCCGCTGTGAGCAAAGGCGAGGAGGATAACATGGCCATCA<br>TCAAGGAGTTCATGAGATTCAAAGTGCACATGGAGGGCAGCGTGAACGGCCACGAGTTTGAGATCGAGGGCGAGGGCGAGGGAA<br>GACCCTACGAGGGCACACAGACCGCCAAGCTGAAAGTGACCAAGGGCGGCCCCCTGCCTTTCGCCTGGGACATCCTGTCCCCTC<br>AGTTCATGTACGGCTCCAAGGCCTACGTGAAGCACCCTGCCGACATCCCTGACTACCTGAAGCTGAGCTTTCCTGAGGGCTTCAAG<br>TGGGAGAGGGTCATGAACTTTGAGGACGGCGGCGTGGTGACAGTGACCCAGGATAGCAGCCTGCAGGATGGCGAGTTTATCTACA<br>AGGTGAAGCTGAGGGGCACCAACTTCCCTAGCGACGGCCCTGTGATGCAGAAGAAGACCATGGGCTGGGAGGCCAGCTCCGAGA<br>GAATGTACCCCGAGGATGGCGCCCTGAAGGGCGAGATCAAGCAGAGGCTGAAGCTGAAGGACGGCGGCCACTACGACGCCGAG<br>GTGAAGACCACATACAAGGCCAAGAAGCCCGTGCAGCTGCCCGGCGCTTACAACGTGAACATCAAGCTGGACATCACCTCCCACA<br>ACGAGGACTACACAATCGTGGAGCAGTACGAGAGGGCCGAGGGCAGGCACTCCACAGGCGGAATGGATGAGCTGTACAAGGGCG<br>GCAGCCTGGTGCCTAGAGGCAGCAGCGCCTGGAGCCACCCTCAGTTCGAGAAGAGCGCCCCTTCCAGACTGGAGGAGGAGCTGA<br>GGAGGAGGCTGACAGAGTAA
TLR3 (A795H)	CTCGAGCCACCATGAGACAGACTTTGCCTTGTATCTACTTTTGGGGGGGCCTTTTGCCCTTTGGGATGCTGTGTGCATCCTCCACC<br>ACCAAGTGCACTGTTAGCCATGAAGTTGCTGACTGCAGCCACCTGAAGTTGACTCAGGTACCCGATGATCTACCCACAAACATAAC<br>AGTGTTGAACCTTACCCATAATCAACTCAGAAGATTACCAGCCGCCAACTTCACAAGGTATAGCCAGCTAACTAGCTTGGATGTAGG<br>ATTTAACACCATCTCAAAACTGGAGCCAGAATTGTGCCAGAAACTTCCCATGTTAAAAGTTTTGAACCTCCAGCACAATGAGCTATCT<br>CAACTTTCTGATAAAACCTTTGCCTTCTGCACGAATTTGACTGAACTCCATCTCATGTCCAACTCAATCCAGAAAATTAAAAATAATCC<br>CTTTGTCAAGCAGAAGAATTTAATCACATTAGATCTGTCTCATAATGGCTTGTCATCTACAAAATTAGGAACTCAGGTTCAGCTGGAA<br>AATCTCCAAGAGCTTCTATTATCAAACAATAAAATTCAAGCGCTAAAAAGTGAAGAACTGGATATCTTTGCCAATTCATCTTTAAAAAA<br>ATTAGAGTTGTCATCGAATCAAATTAAAGAGTTTTCTCCAGGGTGTTTTCACGCAATTGGAAGATTATTTGGCCTCTTTCTGAACAAT<br>GTCCAGCTGGGTCCCAGCCTTACAGAGAAGCTATGTTTGGAATTAGCAAACACAAGCATTCGGAATCTGTCTCTGAGTAACAGCCA<br>GCTGTCCACCACCAGCAATACAACTTTCTTGGGACTAAAGTGGACAAATCTCACTATGCTCGATCTTTCCTACAACAACTTAAATGTG<br>GTTGGTAACGATTCCTTTGCTTGGCTTCCACAACTAGAATATTTCTTCCTAGAGTATAATAATATACAGCATTTGTTTTCTCACTCTTT<br>GCACGGGCTTTTCAATGTGAGGTACCTGAATTTGAAACGGTCTTTTACTAAACAAAGTATTTCCCTTGCCTCACTCCCCAAGATTGAT<br>GATTTTTCTTTTCAGTGGCTAAAATGTTTGGAGCACCTTAACATGGAAGATAATGATATTCCAGGCATAAAAAGCAATATGTTCACAG<br>GATTGATAAACCTGAAATACTTAAGTCTATCCAACTCCTTTACAAGTTTGCGAACTTTGACAAATGAAACATTTGTATCACTTGCTCAT<br>TCTCCCTTACACATACTCAACCTAACCAAGAATAAAATCTCAAAAATAGAGAGTGATGCTTTCTCTTGGTTGGGCCACCTAGAAGTAC<br>TTGACCTGGGCCTTAATGAAATTGGGCAAGAACTCACAGGCCAGGAATGGAGAGGTCTAGAAAATATTTTCGAAATCTATCTTTCCT<br>ACAACAAGTACCTGCAGCTGACTAGGAACTCCTTTGCCTTGGTCCCAAGCCTTCAACGACTGATGCTCCGAAGGGTGGCCCTTAAA<br>AATGTGGATAGCTCTCCTTCACCATTCCAGCCTCTTCGTAACTTGACCATTCTGGATCTAAGCAACAACAACATAGCCAACATAAATG<br>ATGACATGTTGGAGGGTCTTGAGAAACTAGAAATTCTCGATTTGCAGCATAACAACTTAGCACGGCTCTGGAAACACGCAAACCCTG<br>GTGGTCCCATTTATTTCCTAAAGGGTCTGTCTCACCTCCACATCCTTAACTTGGAGTCCAACGGCTTTGACGAGATCCCAGTTGAGG<br>TCTTCAAGGATTTATTTGAACTAAAGATCATCGATTTAGGATTGAATAATTTAAACACACTTCCAGCATCTGTCTTTAATAATCAGGTG<br>TCTCTAAAGTCATTGAACCTTCAGAAGAATCTCATAACATCCGTTGAGAAGAAGGTTTTCGGGCCAGCTTTCAGGAACCTGACTGAG<br>TTAGATATGCGCTTTAATCCCTTTGATTGCACGTGTGAAAGTATTGCCTGGTTTGTTAATTGGATTAACGAGACCCATACCAACATCC<br>CTGAGCTGTCAAGCCACTACCTTTGCAACACTCCACCTCACTATCATGGGTTCCCAGTGAGACTTTTTGATACATCATCTTGCAAAG<br>ACAGTGCCCCCTTTGAACTCTTTTTCATGATCAATACCAGTATCCTGTTGATTTTTATCTTTATTGTACTTCTCATCCACTTTGAGGGC<br>TGGAGGATATCTTTTTATTGGAATGTTTCAGTACATCGAGTTCTTGGTTTCAAAGAAATAGACAGACAGACAGAACAGTTTGAATATG<br>CAGCATATATAATTCATGCCTATAAAGATAAGGATTGGGTCTGGGAACATTTCTCTTCAATGGAAAAGGAAGACCAATCTCTCAAATT<br>TTGTCTGGAAGAAAGGGACTTTGAGCATGGTGTTTTTGAACTAGAAGCAATTGTTAACAGCATCAAAAGAAGCAGAAAAATTATTTTT<br>GTTATAACACACCATCTATTAAAAGACCCATTATGCAAAAGATTCAAGGTACATCATGCAGTTCAACAAGCTATTGAACAAAATCTGG<br>ATTCCATTATATTGGTTTTCCTTGAGGAGATTCCAGATTATAAACTGAACCATGCACTCTGTTTGCGAAGAGGAATGTTTAAATCTCAC<br>TGCATCTTGAACTGGCCAGTTCAGAAAGAACGGATAGGTGCCTTTCGTCATAAATTGCAAGTAGCACTTGGATCCAAAAACTCTGTA<br>CATTCGAATTCTCTGGAGGTGCTGTTTCAGGGCCCTACCGCCGCCGCCGCTGTGAGCAAAGGCGAGGAGGATAACATGGCCATCA<br>TCAAGGAGTTCATGAGATTCAAAGTGCACATGGAGGGCAGCGTGAACGGCCACGAGTTTGAGATCGAGGGCGAGGGCGAGGGAA<br>GACCCTACGAGGGCACACAGACCGCCAAGCTGAAAGTGACCAAGGGCGGCCCCCTGCCTTTCGCCTGGGACATCCTGTCCCCTC<br>AGTTCATGTACGGCTCCAAGGCCTACGTGAAGCACCCTGCCGACATCCCTGACTACCTGAAGCTGAGCTTTCCTGAGGGCTTCAAG<br>TGGGAGAGGGTCATGAACTTTGAGGACGGCGGCGTGGTGACAGTGACCCAGGATAGCAGCCTGCAGGATGGCGAGTTTATCTACA<br>AGGTGAAGCTGAGGGGCACCAACTTCCCTAGCGACGGCCCTGTGATGCAGAAGAAGACCATGGGCTGGGAGGCCAGCTCCGAGA<br>GAATGTACCCCGAGGATGGCGCCCTGAAGGGCGAGATCAAGCAGAGGCTGAAGCTGAAGGACGGCGGCCACTACGACGCCGAG<br>GTGAAGACCACATACAAGGCCAAGAAGCCCGTGCAGCTGCCCGGCGCTTACAACGTGAACATCAAGCTGGACATCACCTCCCACA<br>ACGAGGACTACACAATCGTGGAGCAGTACGAGAGGGCCGAGGGCAGGCACTCCACAGGCGGAATGGATGAGCTGTACAAGGGCG<br>GCAGCCTGGTGCCTAGAGGCAGCAGCGCCTGGAGCCACCCTCAGTTCGAGAAGAGCGCCCCTTCCAGACTGGAGGAGGAGCTGA<br>GGAGGAGGCTGACAGAGTAA

Supplementary Table 7 | ectoTLR3 and mutants for cryo-EM experiments

Protein name	Gene sequences
ectoTLR3	ATGGTAAGCGCTATTGTTTTATATGTGCTTTTGGCGGCGGCGGCGCATTCTGCCTTTGCGGCGGATCCCAAGTGCACTGTTAGCCATGAAGTTGCTGACT<br>GCAGCCACCTGAAGTTGACTCAGGTACCCGATGATCTACCCACAAACATAACAGTGTTGAACCTTACCCATAATCAACTCAGAAGATTACCAGCCGCCAA<br>CTTCACAAGGTATAGCCAGCTAACTAGCTTGGATGTAGGATTTAACACCATCTCAAAACTGGAGCCAGAATTGTGCCAGAAACTTCCCATGTTAAAAGTTT<br>TGAACCTCCAGCACAATGAGCTATCTCAACTTTCTGATAAAACCTTTGCCTTCTGCACGAATTTGACTGAACTCCATCTCATGTCCAACTCAATCCAGAAA<br>ATTAAAAATAATCCCTTTGTCAAGCAGAAGAATTTAATCACATTAGATCTGTCTCATAATGGCTTGTCATCTACAAAATTAGGAACTCAGGTTCAGCTGGAA<br>AATCTCCAAGAGCTTCTATTATCAAACAATAAAATTCAAGCGCTAAAAAGTGAAGAACTGGATATCTTTGCCAATTCATCTTTAAAAAAATTAGAGTTGTCA<br>TCGAATCAAATTAAAGAGTTTTCTCCAGGGTGTTTTCACGCAATTGGAAGATTATTTGGCCTCTTTCTGAACAATGTCCAGCTGGGTCCCAGCCTTACAGA<br>GAAGCTATGTTTGGAATTAGCAAACACAAGCATTCGGAATCTGTCTCTGAGTAACAGCCAGCTGTCCACCACCAGCAATACAACTTTCTTGGGACTAAAG<br>TGGACAAATCTCACTATGCTCGATCTTTCCTACAACAACTTAAATGTGGTTGGTAACGATTCCTTTGCTTGGCTTCCACAACTAGAATATTTCTTCCTAGAG<br>TATAATAATATACAGCATTTGTTTTCTCACTCTTTGCACGGGCTTTTCAATGTGAGGTACCTGAATTTGAAACGGTCTTTTACTAAACAAAGTATTTCCCTTG<br>CCTCACTCCCCAAGATTGATGATTTTTCTTTTCAGTGGCTAAAATGTTTGGAGCACCTTAACATGGAAGATAATGATATTCCAGGCATAAAAAGCAATATGT<br>TCACAGGATTGATAAACCTGAAATACTTAAGTCTATCCAACTCCTTTACAAGTTTGCGAACTTTGACAAATGAAACATTTGTATCACTTGCTCATTCTCCCT<br>TACACATACTCAACCTAACCAAGAATAAAATCTCAAAAATAGAGAGTGATGCTTTCTCTTGGTTGGGCCACCTAGAAGTACTTGACCTGGGCCTTAATGAA<br>ATTGGGCAAGAACTCACAGGCCAGGAATGGAGAGGTCTAGAAAATATTTTCGAAATCTATCTTTCCTACAACAAGTACCTGCAGCTGACTAGGAACTCCT<br>TTGCCTTGGTCCCAAGCCTTCAACGACTGATGCTCCGAAGGGTGGCCCTTAAAAATGTGGATAGCTCTCCTTCACCATTCCAGCCTCTTCGTAACTTGAC<br>CATTCTGGATCTAAGCAACAACAACATAGCCAACATAAATGATGACATGTTGGAGGGTCTTGAGAAACTAGAAATTCTCGATTTGCAGCATAACAACTTAG<br>CACGGCTCTGGAAACACGCAAACCCTGGTGGTCCCATTTATTTCCTAAAGGGTCTGTCTCACCTCCACATCCTTAACTTGGAGTCCAACGGCTTTGACGA<br>GATCCCAGTTGAGGTCTTCAAGGATTTATTTGAACTAAAGATCATCGATTTAGGATTGAATAATTTAAACACACTTCCAGCATCTGTCTTTAATAATCAGGT<br>GTCTCTAAAGTCATTGAACCTTCAGAAGAATCTCATAACATCCGTTGAGAAGAAGGTTTTCGGGCCAGCTTTCAGGAACCTGACTGAGTTAGATATGCGC<br>TTTAATCCCTTTGATTGCACGTGTGAAAGTATTGCCTGGTTTGTTAATTGGATTAACGAGACCCATACCAACATCCCTGAGCTGTCAAGCCACTACCTTTG<br>CAACACTCCACCTCACTATCATGGGTTCCCAGTGAGACTTTTTGATACATCATCTTGCAAAAGCGGCCGCCTGGTTCCGCGTGGTTCGCATCATCATCAT<br>CATCACTGA
NT-mut ectoTLR3	ATGGTAAGCGCTATTGTTTTATATGTGCTTTTGGCGGCGGCGGCGCATTCTGCCTTTGCGGCGGATCCCAAGTGCACTGTTAGCCATGAAGTTGCTGACT<br>GCAGCCACCTGAAGTTGACTCAGGTACCCGATGATCTACCCACAAACATAACAGTGTTGAACCTTACCCATAATCAACTCAGAAGATTACCAGCCGCCAA<br>CTTCACAAGGTATAGCCAGCTAACTAGCTTGGATGTAGGATTTAACACCATCTCAAAACTGGAGCCAGAATTGTGCCAGAAACTTCCCATGTTAAAAGTTT<br>TGAACCTCCAGCACAATGAGCTATCTCAACTTTCTGATgacACCTTTGCCTTCTGCACGAATTTGACTGAACTCCATCTCATGTCCAACTCAATCCAGAAAA<br>TTgatAATAATCCCTTTGTCgatCAGAAGAATTTAATCACATTAGATCTGTCTCATAATGGCTTGTCATCTACAAAATTAGGAACTCAGGTTCAGCTGGAAAAT<br>CTCCAAGAGCTTCTATTATCAAACAATAAAATTCAAGCGCTAAAAAGTGAAGAACTGGATATCTTTGCCAATTCATCTTTAAAAAAATTAGAGTTGTCATCG<br>AATCAAATTAAAGAGTTTTCTCCAGGGTGTTTTCACGCAATTGGAAGATTATTTGGCCTCTTTCTGAACAATGTCCAGCTGGGTCCCAGCCTTACAGAGAA<br>GCTATGTTTGGAATTAGCAAACACAAGCATTCGGAATCTGTCTCTGAGTAACAGCCAGCTGTCCACCACCAGCAATACAACTTTCTTGGGACTAAAGTGG<br>ACAAATCTCACTATGCTCGATCTTTCCTACAACAACTTAAATGTGGTTGGTAACGATTCCTTTGCTTGGCTTCCACAACTAGAATATTTCTTCCTAGAGTAT<br>AATAATATACAGCATTTGTTTTCTCACTCTTTGCACGGGCTTTTCAATGTGAGGTACCTGAATTTGAAACGGTCTTTTACTAAACAAAGTATTTCCCTTGCCT<br>CACTCCCCAAGATTGATGATTTTTCTTTTCAGTGGCTAAAATGTTTGGAGCACCTTAACATGGAAGATAATGATATTCCAGGCATAAAAAGCAATATGTTCA<br>CAGGATTGATAAACCTGAAATACTTAAGTCTATCCAACTCCTTTACAAGTTTGCGAACTTTGACAAATGAAACATTTGTATCACTTGCTCATTCTCCCTTAC<br>ACATACTCAACCTAACCAAGAATAAAATCTCAAAAATAGAGAGTGATGCTTTCTCTTGGTTGGGCCACCTAGAAGTACTTGACCTGGGCCTTAATGAAATT<br>GGGCAAGAACTCACAGGCCAGGAATGGAGAGGTCTAGAAAATATTTTCGAAATCTATCTTTCCTACAACAAGTACCTGCAGCTGACTAGGAACTCCTTTG<br>CCTTGGTCCCAAGCCTTCAACGACTGATGCTCCGAAGGGTGGCCCTTAAAAATGTGGATAGCTCTCCTTCACCATTCCAGCCTCTTCGTAACTTGACCAT<br>TCTGGATCTAAGCAACAACAACATAGCCAACATAAATGATGACATGTTGGAGGGTCTTGAGAAACTAGAAATTCTCGATTTGCAGCATAACAACTTAGCA<br>CGGCTCTGGAAACACGCAAACCCTGGTGGTCCCATTTATTTCCTAAAGGGTCTGTCTCACCTCCACATCCTTAACTTGGAGTCCAACGGCTTTGACGAGA<br>TCCCAGTTGAGGTCTTCAAGGATTTATTTGAACTAAAGATCATCGATTTAGGATTGAATAATTTAAACACACTTCCAGCATCTGTCTTTAATAATCAGGTGT<br>CTCTAAAGTCATTGAACCTTCAGAAGAATCTCATAACATCCGTTGAGAAGAAGGTTTTCGGGCCAGCTTTCAGGAACCTGACTGAGTTAGATATGCGCTTT<br>AATCCCTTTGATTGCACGTGTGAAAGTATTGCCTGGTTTGTTAATTGGATTAACGAGACCCATACCAACATCCCTGAGCTGTCAAGCCACTACCTTTGCAA<br>CACTCCACCTCACTATCATGGGTTCCCAGTGAGACTTTTTGATACATCATCTTGCAAAAGCGGCCGCCTGGTTCCGCGTGGTTCGCATCATCATCATCAT<br>CACTGA
CT-mut ectoTLR3	ATGGTAAGCGCTATTGTTTTATATGTGCTTTTGGCGGCGGCGGCGCATTCTGCCTTTGCGGCGGATCCCAAATGCACCGTAAGTCACGAAGTGGCAGAC<br>TGTTCTCATCTGAAACTGACTCAAGTGCCTGATGACTTGCCTACCAATATTACGGTGTTGAATCTCACTCATAACCAGCTTCGTCGCCTACCTGCCGCTAA<br>TTTTACGAGATACTCGCAACTCACTAGCTTGGATGTGGGTTTTAACACTATAAGTAAACTCGAACCAGAACTTTGTCAAAAGCTGCCCATGCTTAAAGTCC<br>TTAATTTGCAGCATAATGAGCTTTCCCAACTTAGTGACAAGACTTTTGCTTTCTGTACTAATTTGACGGAACTCCATCTTATGTCGAACTCGATCCAGAAAA<br>TTAAGAACAATCCGTTTGTGAAACAGAAGAATTTGATCACATTGGATCTCTCCCACAACGGCTTGAGCTCCACTAAACTGGGAACCCAAGTCCAACTGGA<br>GAACCTACAGGAATTGCTGCTGTCGAATAATAAAATTCAAGCTCTAAAATCAGAGGAGCTAGACATCTTCGCCAATTCTTCCCTCAAGAAACTTGAACTGT<br>CCTCAAATCAAATCAAGGAATTCTCACCCGGATGTTTCCATGCAATCGGTCGTCTGTTTGGTTTGTTTCTTAATAACGTGCAGCTTGGACCCTCATTGACC<br>GAGAAACTCTGTCTGGAATTGGCCAATACCTCTATACGCAACCTATCCTTGAGCAATTCGCAACTATCGACTACATCGAATACCACCTTTCTGGGTCTGAA<br>ATGGACGAATCTGACCATGCTGGACTTGTCTTATAACAATCTCAACGTCGTCGGTAATGACTCTTTTGCTTGGCTGCCTCAGTTGGAGTATTTCTTTCTCG<br>AATATAATAATATCCAGCATCTATTTTCCCATTCTCTCCACGGACTATTTAACGTGAGGTATCTGAACTTGAAAAGGAGTTTCACGAAACAGTCAATCTCCC<br>TCGCTTCGCTCCCTAAAATTGATGACTTTTCCTTTCAATGGTTGAAATGCCTGGAACACCTAAATATGGAAGATAACGACATTCCCGGTATTAAAAGTAAT<br>ATGTTCACGGGCCTCATTAATTTGAAGTATCTTTCGCTCTCCAACTCCTTCACCAGTCTGCGAACCCTTACCAATGAAACCTTTGTCTCACTCGCCCACTC<br>ACCCCTGCATATTTTGAATCTCACCAAGAATAAAATTTCCAAAATTGAGTCAGACGCTTTTAGCTGGCTTGGCCACCTGGAAGTACTCGATCTCGGCCTCA<br>ATGAGATTGGCCAAGAATTGACCGGCCAGGAATGGCGCGGTCTTGAGAATATTTTCGAAATCTATCTGTCTTACAATAAGTATCTCCAATTGACTCGTAAC<br>TCTTTTGCCCTGGTGCCGTCCCTACAGCGTCTCATGTTGAGGCGTGTTGCGCTAAAGAATGTTGATTCCAGTCCTAGCCCTTTTCAACCTTTGAGAAATCT<br>GACAATCCTTGACCTCAGCAACAACAACATCGCCAACATCAATAAGAAAATGCTAAAAGGTCTGGAAAAGCTCGAAATCTTGGACCTACAGCATAACAAT<br>TTGGCCCGTCTATGGAAACACGCCAATCCTGGTGGACCCATTTATTTTCTCAAGGGACTGTCTCATCTCCACATACTAAACCTTGAATCGAACGGATTTGA<br>TGAAATCCCCGTTGAGGTGTTTAAGGACCTCTTTGAGTTGAAGATCATTGATCTGGGACTTAATAATCTGAACACCCTTCCGGCTAGTGTCTTTAATAATC<br>AAGTGTCCCTGAAATCACTTAACTTGCAGAAGAACTTGATAACTAGCGTCGAGAAGAAAGTCTTTGGACCGGCTTTTCGCAACCTAACGGAACTCGACAT<br>GCGTTTTAACCCATTCGACTGCACCTGCGAATCTATTGCATGGTTCGTGAACTGGATCAATGAGACCCATACCAACATTCCAGAATTGTCATCGCACTAC<br>CTGTGTAATACCCCACCCCATTACCACGGATTTCCTGTCAGGCTTTTCGATACTTCCAGCTGCAAACATCACCACCATCATCATTGA

Supplementary Table 8 | mAb12 gene sequences

Protein name	Gene sequences
scFv form of mAb12	ATGGTAAGCGCTATTGTTTTATATGTGCTTTTGGCGGCGGCGGCGCATTCTGCCTTTGCGGCGGATCCCCAAGTACAACTACA<br>GCAGTCGGGTCCTGGCCTGGTTAAACCATCCCAGACGCTTAGTTTGACTTGTGCCATATCTGGTGACAGCGTTTCATCGAATA<br>GCGCTGCGTGGGGCTGGATTCGCCAATCACCAGGTAGAGGACTCGAGTGGTTAGGTATTATACAGAAGCGATCCAAGTGGTA<br>TAATAATTATGCAGTGTCCGTTAAATCCCGTATAACCATTAATCCAGACACGTCCAAGAATCAATTCAGTCTCCAGCTCAACAG<br>TGTGACCCCCGAAGATACTGCTGTTTATTATTGTGCTCGATACAGTTACCCTTTCTACAGCATAGATTATTGGGGCCAGGGTAC<br>TCTGGTGACAGTGAGTTCAGGTGGCGGCGGATCAGGTGGTGGTGGATCGGGTGGAGGAGGTAGCGGTGGCGGCGGTAGTC<br>AGTCAGTCCTTACCCAACCCCCGTCCGTTTCAGTTGCTCCAGGCCAAACAGCACGCATTTCATGTTCTGGAGATAATATCGGA<br>AGCTATTATGTGCACTGGTACCAGCAAAAGCCAGGTCAAGCGCCCGTCTTAGTAATATACGAGGATTCCGAGAGGCCGTCAG<br>GTATACCAGAACGATTTAGCGGATCAAACTCAGGCAATACAGCAACTCTAACCATCAGCGGCACACAAGCTGAGGATGAAGCA<br>GACTATTATTGTTCGAGCTATGACGATCCTAATTTTCAAGTCTTTGGTGGTGGCACAAAACTGACAGTGCTTGGTCATCATCAT<br>CATCACCACCACCACTAA
Fab form of mAb12	Heavy chain:<br>CTCGAGCCACCATGGAGTTTGGGCTTTCCTGGGTGTTTCTTGTTGCTTTACTGCGGGGCGTACAATGTCAAGTACAACTGCAG<br>CAATCTGGTCCAGGCCTGGTTAAACCTAGCCAAACTTTGTCTCTGACCTGCGCAATCAGCGGCGACTCTGTGTCATCAAACTC<br>AGCTGCGTGGGGATGGATCAGGCAATCTCCCGGCCGAGGGCTCGAATGGCTCGGCATTATCCAAAAGAGAAGCAAGTGGTA<br>CAATAACTATGCGGTTAGCGTAAAATCTAGGATCACTATTAATCCTGATACATCCAAGAATCAGTTTTCCCTGCAATTAAACAGT<br>GTGACACCCGAAGACACCGCCGTTTACTATTGTGCGCGATACTCTTATCCCTTTTACAGCATTGATTACTGGGGCCAAGGAAC<br>CTTGGTGACCGTGTCAAGCGCCTCTACCAAAGGGCCAAGCGTGTTTCCGTTAGCTCCTTGCAGCAGATCTACAAGTGAATCTA<br>CTGCCGCATTGGGATGCCTCGTGAAGGACTACTTTCCGGAACCGGTGACCGTCTCATGGAACAGTGGCGCACTCACCTCTGG<br>TGTTCACACCTTTCCGGCTGTGTTACAATCCTCAGGTTTGTATTCCTTGAGTAGCGTCGTCACAGTGCCATCTTCCTCACTGGG<br>AACTAAAACTTATACCTGCAATGTGGATCACAAACCATCCAATACGAAAGTTGACAAAAGGGTTCATCATCACCATCACCATTC<br>GAATTCTCTGGAGGTGCTGTTTCAGGGCCCTACCGCCGCCGCCGCTGTGAGCAAAGGCGAGGAGGATAACATGGCCATCAT<br>CAAGGAGTTCATGAGATTCAAAGTGCACATGGAGGGCAGCGTGAACGGCCACGAGTTTGAGATCGAGGGCGAGGGCGAGGG<br>AAGACCCTACGAGGGCACACAGACCGCCAAGCTGAAAGTGACCAAGGGCGGCCCCCTGCCTTTCGCCTGGGACATCCTGTC<br>CCCTCAGTTCATGTACGGCTCCAAGGCCTACGTGAAGCACCCTGCCGACATCCCTGACTACCTGAAGCTGAGCTTTCCTGAG<br>GGCTTCAAGTGGGAGAGGGTCATGAACTTTGAGGACGGCGGCGTGGTGACAGTGACCCAGGATAGCAGCCTGCAGGATGGC<br>GAGTTTATCTACAAGGTGAAGCTGAGGGGCACCAACTTCCCTAGCGACGGCCCTGTGATGCAGAAGAAGACCATGGGCTGG<br>GAGGCCAGCTCCGAGAGAATGTACCCCGAGGATGGCGCCCTGAAGGGCGAGATCAAGCAGAGGCTGAAGCTGAAGGACGG<br>CGGCCACTACGACGCCGAGGTGAAGACCACATACAAGGCCAAGAAGCCCGTGCAGCTGCCCGGCGCTTACAACGTGAACAT<br>CAAGCTGGACATCACCTCCCACAACGAGGACTACACAATCGTGGAGCAGTACGAGAGGGCCGAGGGCAGGCACTCCACAGG<br>CGGAATGGATGAGCTGTACAAGGGCGGCAGCCTGGTGCCTAGAGGCAGCAGCGCCTGGAGCCACCCTCAGTTCGAGAAGA<br>GCGCCCCTTCCAGACTGGAGGAGGAGCTGAGGAGGAGGCTGACAGAGTAA<br><br>Light chain:<br>CTCGAGCCACCATGGAATTTGGACTGAGCTGGGTGTTCTTGGTGGCCCTCTTACGCGGTGTCCAGTGCGAGAGCGTGCTTAC<br>GCAACCACCAAGTGTATCTGTGGCTCCGGGGCAAACAGCACGCATAAGCTGTAGTGGCGATAATATCGGATCATACTACGTG<br>CATTGGTATCAGCAGAAACCTGGCCAAGCTCCAGTTCTTGTGATATACGAAGACTCTGAACGGCCATCTGGGATCCCTGAAAG<br>GTTTAGCGGTAGCAACAGCGGAAACACAGCCACCCTGACAATCAGCGGGACGCAAGCCGAAGACGAGGCGGACTATTATTG<br>CAGTTCCTACGATGATCCTAATTTTCAAGTGTTCGGCGGTGGCACCAAACTGACTGTGCTTGGTCAACCTAAGGCAGCTCCAA<br>GCGTCACCCTCTTTCCACCCAGCAGCGAGGAGCTTCAAGCAAACAAAGCAACGCTCGTGTGTCTGATTTCCGACTTCTACCCT<br>GGAGCAGTGACTGTTGCCTGGAAAGCTGATTCAAGCCCCGTGAAGGCTGGAGTGGAAACCACTACCCCGTCAAAGCAGAGC<br>AATAACAAGTACGCCGCATCTTCCTACCTTTCCCTCACCCCTGAGCAGTGGAAGTCTCACAGGTCCTACTCATGCCAAGTTAC<br>ACACGAGGGTTCTACTGTCGAAAAGACGGTCGCCCCTACGGAATGTAGCTCGAATTCTTTGGAAGTTTTGTTTCAAGGTCCAA<br>CTGCTGCCGCCGCTGTGAGCAAGGGCGAGGAGCTGTTCACCGGGGTGGTGCCCATCCTGGTCGAGCTGGACGGCGACGTA<br>AACGGCCACAAGTTCAGCGTGTCCGGCGAGGGCGAGGGCGATGCCACCTACGGCAAGCTGACCCTGAAGTTCATCTGCACC<br>ACCGGCAAGCTGCCCGTGCCCTGGCCCACCCTCGTGACCACCCTGACCTACGGCGTGCAGTGCTTCAGCCGCTACCCCGAC<br>CACATGAAGCAGCACGACTTCTTCAAGTCCGCCATGCCCGAAGGCTACGTCCAGGAGCGCACCATCTTCTTCAAGGACGACG<br>GCAACTACAAGACCCGCGCCGAGGTGAAGTTCGAGGGCGACACCCTGGTGAACCGCATCGAGCTGAAGGGCATCGACTTCA<br>AGGAGGACGGCAACATCCTGGGGCACAAGCTGGAGTACAACTACAACAGCCACAACGTCTATATCATGGCCGACAAGCAGAA<br>GAACGGCATCAAGGTGAACTTCAAGATCCGCCACAACATCGAGGACGGCAGCGTGCAGCTCGCCGACCACTACCAGCAGAA<br>CACCCCCATCGGCGACGGCCCCGTGCTGCTGCCCGACAACCACTACCTGAGCACCCAGTCCAAGCTGAGCAAAGACCCCAA<br>CGAGAAGCGCGATCACATGGTCCTGCTGGAGTTCGTGACCGCCGCCGGGATCACTCTCGGCATGGACGAGCTGTACAAGTC<br>CGGAGGTGGTCACCATCACCATCACCACCATCACCATCACTAA

Supplementary Table 9 | TLR3 gene sequences for activity assays

Protein name	Gene sequences
TLR3	GGATCCACCATGAGGCAGACACTGCCCTGTATCTACTTTTGGGGCGGCCTGCTGCCTTTCGGCATGCTGTGCGCCAGCGATTACAAGGA<br>TGATGATGACAAGAGCACCACAAAGTGCACCGTGAGCCACGAGGTGGCCGACTGCAGCCACCTGAAGCTGACCCAGGTGCCCGACGAC<br>CTGCCTACAAACATCACCGTGCTGAATCTGACACACAATCAGCTGAGAAGACTGCCCGCCGCCAACTTCACAAGGTACTCCCAGCTGACA<br>AGCCTGGATGTGGGCTTCAACACCATCAGCAAGCTGGAGCCTGAGCTGTGTCAGAAGCTGCCCATGCTGAAGGTGCTGAATCTCCAGCA<br>CAATGAGCTGAGCCAGCTGTCCGACAAGACATTTGCCTTTTGTACAAACCTGACCGAGCTGCACCTGATGAGCAATAGCATCCAGAAGAT<br>CAAGAATAACCCTTTCGTGAAGCAGAAGAATCTGATCACCCTGGATCTGAGCCACAATGGCCTGAGCTCCACAAAGCTGGGCACACAGGT<br>GCAGCTGGAGAACCTGCAGGAGCTGCTGCTGTCCAATAATAAGATCCAGGCCCTGAAGAGCGAGGAGCTGGACATCTTCGCCAACTCCT<br>CCCTGAAGAAGCTGGAGCTGAGCAGCAACCAGATCAAGGAGTTCAGCCCCGGCTGCTTTCACGCCATCGGCAGGCTGTTTGGCCTGTTC<br>CTGAACAATGTGCAGCTGGGCCCCTCCCTGACAGAGAAGCTGTGTCTGGAGCTGGCCAATACATCCATCAGGAATCTGTCCCTGTCCAAT<br>AGCCAGCTGAGCACCACAAGCAACACCACATTCCTGGGCCTGAAGTGGACCAATCTGACAATGCTGGACCTGTCCTACAACAATCTGAAC<br>GTGGTGGGCAACGACAGCTTTGCCTGGCTGCCCCAGCTGGAGTACTTCTTTCTGGAGTACAACAATATCCAGCACCTGTTTTCCCACTCC<br>CTGCACGGCCTGTTCAACGTGAGATACCTGAACCTGAAGAGGAGCTTTACCAAGCAGTCCATCTCCCTGGCCTCCCTGCCTAAGATCGAT<br>GATTTTTCCTTCCAGTGGCTGAAGTGTCTGGAGCACCTGAACATGGAGGACAACGATATCCCCGGCATCAAGTCCAATATGTTCACAGGC<br>CTGATCAATCTGAAGTACCTGTCCCTGAGCAATTCCTTCACATCCCTGAGGACCCTGACCAACGAGACCTTTGTGAGCCTGGCCCACTCC<br>CCCCTGCACATCCTGAACCTGACAAAGAACAAGATCAGCAAGATCGAGAGCGATGCCTTCAGCTGGCTGGGCCACCTGGAGGTGCTGGA<br>CCTGGGCCTGAACGAGATCGGCCAGGAGCTGACAGGCCAGGAGTGGAGGGGCCTGGAGAATATCTTCGAGATCTACCTGAGCTACAACA<br>AGTACCTGCAGCTGACCAGAAACTCCTTCGCCCTGGTGCCCTCCCTGCAGAGGCTGATGCTGAGGAGGGTGGCCCTGAAGAACGTGGAC<br>AGCAGCCCCAGCCCCTTCCAGCCTCTGAGGAACCTGACAATCCTGGACCTGAGCAATAATAACATCGCCAACATCAACGATGACATGCTG<br>GAGGGCCTGGAGAAGCTGGAGATCCTGGATCTGCAGCACAACAACCTGGCCAGGCTGTGGAAGCACGCCAATCCCGGCGGCCCCATCT<br>ACTTTCTGAAGGGCCTGAGCCACCTGCACATCCTCAATCTGGAGAGCAACGGCTTTGACGAGATCCCTGTGGAGGTGTTTAAGGATCTGT<br>TCGAGCTGAAGATCATCGATCTGGGCCTGAATAACCTGAACACACTGCCTGCCAGCGTGTTTAATAACCAGGTGAGCCTGAAGTCCCTGA<br>ACCTGCAGAAGAATCTCATCACCAGCGTGGAGAAGAAGGTGTTCGGCCCTGCCTTCAGAAATCTGACAGAGCTGGACATGAGATTCAATC<br>CTTTCGACTGCACATGTGAGAGCATCGCCTGGTTCGTGAACTGGATCAATGAGACCCACACCAATATCCCTGAGCTGAGCTCCCACTACC<br>TGTGCAACACCCCCCCCCACTACCACGGCTTCCCTGTGAGACTGTTCGACACCAGCTCCTGTAAGGACTCCGCCCCTTTCGAGCTGTTTT<br>TCATGATCAATACCTCCATCCTGCTGATCTTTATCTTCATCGTGCTGCTGATCCACTTTGAGGGCTGGAGGATCAGCTTTTACTGGAACGT<br>GTCCGTGCACAGAGTGCTGGGCTTCAAGGAGATCGATAGGCAGACAGAGCAGTTCGAGTACGCCGCCTACATCATCCACGCCTACAAGG<br>ACAAGGATTGGGTGTGGGAGCACTTCTCCAGCATGGAGAAGGAGGACCAGAGCCTGAAGTTTTGTCTGGAGGAGAGGGACTTTGAGGCC<br>GGCGTGTTTGAGCTGGAGGCCATCGTGAACAGCATCAAGAGGTCCAGGAAGATCATCTTCGTGATCACCCACCACCTGCTGAAGGACCC<br>TCTGTGTAAGAGGTTCAAGGTGCACCACGCCGTGCAGCAGGCCATCGAGCAGAACCTGGATAGCATCATCCTGGTGTTCCTGGAGGAGA<br>TCCCCGACTACAAGCTGAACCACGCCCTGTGCCTGAGAAGGGGCATGTTCAAGAGCCACTGCATCCTGAATTGGCCCGTGCAGAAGGAG<br>AGAATCGGCGCCTTCAGACACAAGCTGCAGGTGGCCCTGGGCTCCAAGAACAGCGTGCACTAACTCGAG
NT-mut TLR3	GGATCCACCATGAGGCAGACACTGCCCTGTATCTACTTTTGGGGCGGCCTGCTGCCTTTCGGCATGCTGTGCGCCAGCGATTACAAGGA<br>TGATGATGACAAGAGCACCACAAAGTGCACTGTTAGCCATGAAGTTGCTGACTGCAGCCACCTGAAGTTGACTCAGGTACCCGATGATCT<br>ACCCACAAACATAACAGTGTTGAACCTTACCCATAATCAACTCAGAAGATTACCAGCCGCCAACTTCACAAGGTATAGCCAGCTAACTAGC<br>TTGGATGTAGGATTTAACACCATCTCAAAACTGGAGCCAGAATTGTGCCAGAAACTTCCCATGTTAAAAGTTTTGAACCTCCAGCACAATGA<br>GCTATCTCAACTTTCTGATGACACCTTTGCCTTCTGCACGAATTTGACTGAACTCCATCTCATGTCCAACTCAATCCAGAAAATTGATAATAA<br>TCCCTTTGTCGATCAGAAGAATTTAATCACATTAGATCTGTCTCATAATGGCTTGTCATCTACAAAATTAGGAACTCAGGTTCAGCTGGAAA<br>ATCTCCAAGAGCTTCTATTATCAAACAATAAAATTCAAGCGCTAAAAAGTGAAGAACTGGATATCTTTGCCAATTCATCTTTAAAAAAATTAG<br>AGTTGTCATCGAATCAAATTAAAGAGTTTTCTCCAGGGTGTTTTCACGCAATTGGAAGATTATTTGGCCTCTTTCTGAACAATGTCCAGCTG<br>GGTCCCAGCCTTACAGAGAAGCTATGTTTGGAATTAGCAAACACAAGCATTCGGAATCTGTCTCTGAGTAACAGCCAGCTGTCCACCACCA<br>GCAATACAACTTTCTTGGGACTAAAGTGGACAAATCTCACTATGCTCGATCTTTCCTACAACAACTTAAATGTGGTTGGTAACGATTCCTTT<br>GCTTGGCTTCCACAACTAGAATATTTCTTCCTAGAGTATAATAATATACAGCATTTGTTTTCTCACTCTTTGCACGGGCTTTTCAATGTGAGG<br>TACCTGAATTTGAAACGGTCTTTTACTAAACAAAGTATTTCCCTTGCCTCACTCCCCAAGATTGATGATTTTTCTTTTCAGTGGCTAAAATGT<br>TTGGAGCACCTTAACATGGAAGATAATGATATTCCAGGCATAAAAAGCAATATGTTCACAGGATTGATAAACCTGAAATACTTAAGTCTATC<br>CAACTCCTTTACAAGTTTGCGAACTTTGACAAATGAAACATTTGTATCACTTGCTCATTCTCCCTTACACATACTCAACCTAACCAAGAATAA<br>AATCTCAAAAATAGAGAGTGATGCTTTCTCTTGGTTGGGCCACCTAGAAGTACTTGACCTGGGCCTTAATGAAATTGGGCAAGAACTCACA<br>GGCCAGGAATGGAGAGGTCTAGAAAATATTTTCGAAATCTATCTTTCCTACAACAAGTACCTGCAGCTGACTAGGAACTCCTTTGCCTTGG<br>TCCCAAGCCTTCAACGACTGATGCTCCGAAGGGTGGCCCTTAAAAATGTGGATAGCTCTCCTTCACCATTCCAGCCTCTTCGTAACTTGAC<br>CATTCTGGATCTAAGCAACAACAACATAGCCAACATAAATGATGACATGTTGGAGGGTCTTGAGAAACTAGAAATTCTCGATTTGCAGCATA<br>ACAACTTAGCACGGCTCTGGAAACACGCAAACCCTGGTGGTCCCATTTATTTCCTAAAGGGTCTGTCTCACCTCCACATCCTTAACTTGGA<br>GTCCAACGGCTTTGACGAGATCCCAGTTGAGGTCTTCAAGGATTTATTTGAACTAAAGATCATCGATTTAGGATTGAATAATTTAAACACAC<br>TTCCAGCATCTGTCTTTAATAATCAGGTGTCTCTAAAGTCATTGAACCTTCAGAAGAATCTCATAACATCCGTTGAGAAGAAGGTTTTCGGG<br>CCAGCTTTCAGGAACCTGACTGAGTTAGATATGCGCTTTAATCCCTTTGATTGCACGTGTGAAAGTATTGCCTGGTTTGTTAATTGGATTAA<br>CGAGACCCATACCAACATCCCTGAGCTGTCAAGCCACTACCTTTGCAACACTCCCCCCCACTACCACGGCTTCCCTGTGAGACTGTTCGA<br>CACCAGCTCCTGTAAGGACTCCGCCCCTTTCGAGCTGTTTTTCATGATCAATACCTCCATCCTGCTGATCTTTATCTTCATCGTGCTGCTG<br>ATCCACTTTGAGGGCTGGAGGATCAGCTTTTACTGGAACGTGTCCGTGCACAGAGTGCTGGGCTTCAAGGAGATCGATAGGCAGACAGA<br>GCAGTTCGAGTACGCCGCCTACATCATCCACGCCTACAAGGACAAGGATTGGGTGTGGGAGCACTTCTCCAGCATGGAGAAGGAGGACC<br>AGAGCCTGAAGTTTTGTCTGGAGGAGAGGGACTTTGAGGCCGGCGTGTTTGAGCTGGAGGCCATCGTGAACAGCATCAAGAGGTCCAGG<br>AAGATCATCTTCGTGATCACCCACCACCTGCTGAAGGACCCTCTGTGTAAGAGGTTCAAGGTGCACCACGCCGTGCAGCAGGCCATCGA<br>GCAGAACCTGGATAGCATCATCCTGGTGTTCCTGGAGGAGATCCCCGACTACAAGCTGAACCACGCCCTGTGCCTGAGAAGGGGCATGT<br>TCAAGAGCCACTGCATCCTGAATTGGCCCGTGCAGAAGGAGAGAATCGGCGCCTTCAGACACAAGCTGCAGGTGGCCCTGGGCTCCAAG<br>AACAGCGTGCACTAACTCGAG
CT-mut TLR3	GGATCCACCATGAGGCAGACACTGCCCTGTATCTACTTTTGGGGCGGCCTGCTGCCTTTCGGCATGCTGTGCGCCAGCGATTACAAGGA<br>TGATGATGACAAGAGCACCACAAAGTGCACCGTGAGCCACGAGGTGGCCGACTGCAGCCACCTGAAGCTGACCCAGGTGCCCGACGAC<br>CTGCCTACAAACATCACCGTGCTGAATCTGACACACAATCAGCTGAGAAGACTGCCCGCCGCCAACTTCACAAGGTACTCCCAGCTGACA<br>AGCCTGGATGTGGGCTTCAACACCATCAGCAAGCTGGAGCCTGAGCTGTGTCAGAAGCTGCCCATGCTGAAGGTGCTGAATCTCCAGCA<br>CAATGAGCTGAGCCAGCTGTCCGACAAGACATTTGCCTTTTGTACAAACCTGACCGAGCTGCACCTGATGAGCAATAGCATCCAGAAGAT<br>CAAGAATAACCCTTTCGTGAAGCAGAAGAATCTGATCACCCTGGATCTGAGCCACAATGGCCTGAGCTCCACAAAGCTGGGCACACAGGT<br>GCAGCTGGAGAACCTGCAGGAGCTGCTGCTGTCCAATAATAAGATCCAGGCCCTGAAGAGCGAGGAGCTGGACATCTTCGCCAACTCCT<br>CCCTGAAGAAGCTGGAGCTGAGCAGCAACCAGATCAAGGAGTTCAGCCCCGGCTGCTTTCACGCCATCGGCAGGCTGTTTGGCCTGTTC<br>CTGAACAATGTGCAGCTGGGCCCCTCCCTGACAGAGAAGCTGTGTCTGGAGCTGGCCAATACATCCATCAGGAATCTGTCCCTGTCCAAT<br>AGCCAGCTGAGCACCACAAGCAACACCACATTCCTGGGCCTGAAGTGGACCAATCTGACAATGCTGGACCTGTCCTACAACAATCTGAAC<br>GTGGTGGGCAACGACAGCTTTGCCTGGCTGCCCCAGCTGGAGTACTTCTTTCTGGAGTACAACAATATCCAGCACCTGTTTTCCCACTCC<br>CTGCACGGCCTGTTCAACGTGAGATACCTGAACCTGAAGAGGAGCTTTACCAAGCAGTCCATCTCCCTGGCCTCCCTGCCTAAGATCGAT<br>GATTTTTCCTTCCAGTGGCTGAAGTGTCTGGAGCACCTGAACATGGAGGACAACGATATCCCCGGCATCAAGTCCAATATGTTCACAGGC<br>CTGATCAATCTGAAGTACCTGTCCCTGAGCAATTCCTTCACATCCCTGAGGACCCTGACCAACGAGACCTTTGTGAGCCTGGCCCACTCC<br>CCCCTGCACATCCTGAACCTGACAAAGAACAAGATCAGCAAGATCGAGAGCGATGCCTTCAGCTGGCTGGGCCACCTGGAGGTGCTGGA<br>CCTGGGCCTGAACGAGATCGGCCAGGAGCTGACAGGCCAGGAGTGGAGGGGCCTGGAGAATATCTTCGAGATCTACCTGAGCTACAACA<br>AGTACCTGCAGCTGACCAGAAACTCCTTCGCCCTGGTGCCCTCCCTGCAGAGGCTGATGCTGAGGAGGGTGGCCCTGAAGAATGTTGAT<br>TCCAGTCCTAGCCCTTTTCAACCTTTGAGAAATCTGACAATCCTTGACCTCAGCAACAACAACATCGCCAACATCAATAAGAAAATGCTAAA<br>AGGTCTGGAAAAGCTCGAAATCTTGGACCTACAGCATAACAATTTGGCCCGTCTATGGAAACACGCCAATCCTGGTGGACCCATTTATTTT<br>CTCAAGGGACTGTCTCATCTCCACATACTAAACCTTGAATCGAACGGATTTGATGAAATCCCCGTTGAGGTGTTTAAGGACCTCTTTGAGC<br>TGAAGATCATCGATCTGGGCCTGAATAACCTGAACACACTGCCTGCCAGCGTGTTTAATAACCAGGTGAGCCTGAAGTCCCTGAACCTGC<br>AGAAGAATCTCATCACCAGCGTGGAGAAGAAGGTGTTCGGCCCTGCCTTCAGAAATCTGACAGAGCTGGACATGAGATTCAATCCTTTCG<br>ACTGCACATGTGAGAGCATCGCCTGGTTCGTGAACTGGATCAATGAGACCCACACCAATATCCCTGAGCTGAGCTCCCACTACCTGTGCA<br>ACACCCCCCCCCACTACCACGGCTTCCCTGTGAGACTGTTCGACACCAGCTCCTGTAAGGACTCCGCCCCTTTCGAGCTGTTTTTCATGA<br>TCAATACCTCCATCCTGCTGATCTTTATCTTCATCGTGCTGCTGATCCACTTTGAGGGCTGGAGGATCAGCTTTTACTGGAACGTGTCCGT<br>GCACAGAGTGCTGGGCTTCAAGGAGATCGATAGGCAGACAGAGCAGTTCGAGTACGCCGCCTACATCATCCACGCCTACAAGGACAAGG<br>ATTGGGTGTGGGAGCACTTCTCCAGCATGGAGAAGGAGGACCAGAGCCTGAAGTTTTGTCTGGAGGAGAGGGACTTTGAGGCCGGCGTG<br>TTTGAGCTGGAGGCCATCGTGAACAGCATCAAGAGGTCCAGGAAGATCATCTTCGTGATCACCCACCACCTGCTGAAGGACCCTCTGTGT<br>AAGAGGTTCAAGGTGCACCACGCCGTGCAGCAGGCCATCGAGCAGAACCTGGATAGCATCATCCTGGTGTTCCTGGAGGAGATCCCCGA<br>CTACAAGCTGAACCACGCCCTGTGCCTGAGAAGGGGCATGTTCAAGAGCCACTGCATCCTGAATTGGCCCGTGCAGAAGGAGAGAATCG<br>GCGCCTTCAGACACAAGCTGCAGGTGGCCCTGGGCTCCAAGAACAGCGTGCACTAACTCGAG

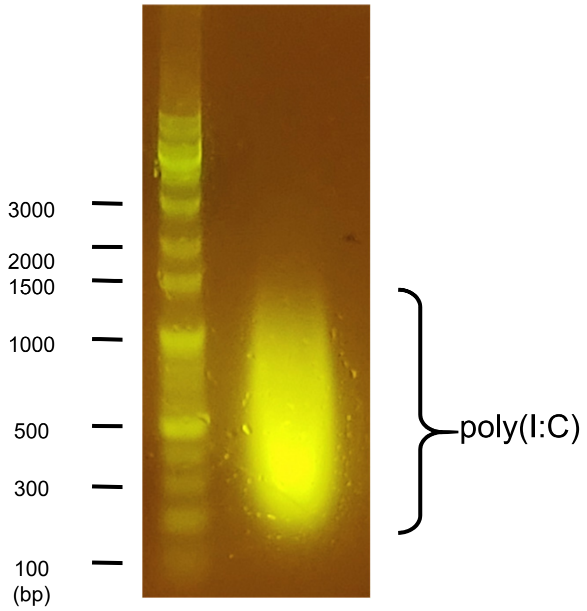

Supplementary Fig. 1 | Size distribution of poly(I:C). Gel electrophoresis image of poly(I:C) used for the TLR3-poly(I:C) complex formation. The size of the poly(I:C) varies from about 200 to 1500 bp and the average size is ~400 bp. The experiment was performed in duplicate and a representative gel image is shown. Source data are provided as a Source Data file.

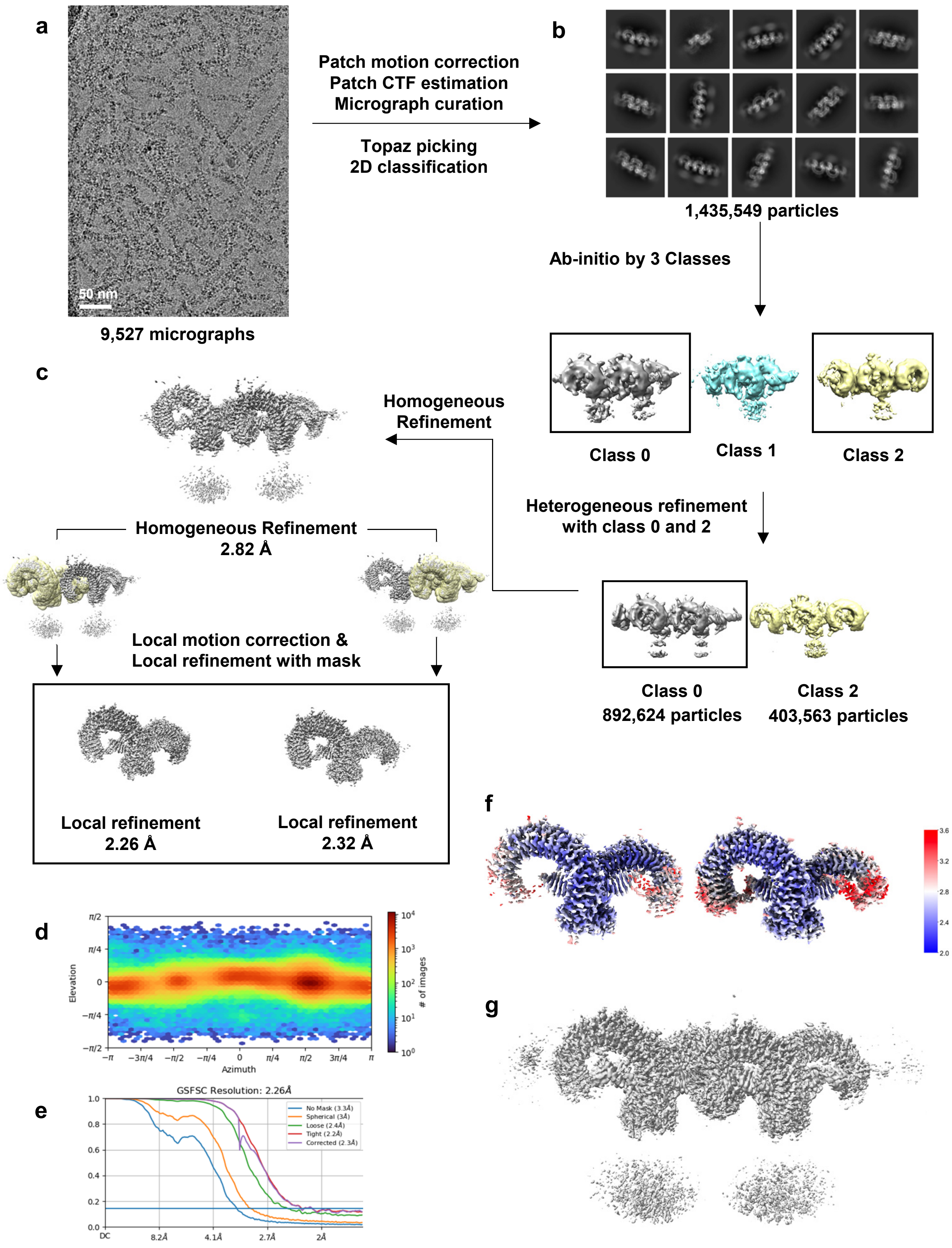
a
50 nm
9,527 micrographs
Patch motion correction
Patch CTF estimation
Micrograph curation
Topaz picking
2D classification
b
1,435,549 particles
Ab-initio by 3 Classes
Class 0
Class 1
Class 2
Heterogeneous refinement
with class 0 and 2
Class 0
892,624 particles
Class 2
403,563 particles
Homogeneous
Refinement
c
Homogeneous Refinement
2.82 Å
Local motion correction &
Local refinement with mask
Local refinement
2.26 Å
Local refinement
2.32 Å
d
Elevation
Azimuth
# of images
e
GSFSC Resolution: 2.26Å
No Mask (3.3Å)
Spherical (3Å)
Loose (2.4Å)
Tight (2.2Å)
Corrected (2.3Å)
f
g

Supplementary Fig. 2 | Cryo-EM data processing of the TLR3-poly(I:C) complex. **a** Representative micrograph of the TLR3-poly(I:C) complex. **b** Selected 2D class average images of the TLR3-poly(I:C) complex. **c** Summary of cryo-EM data processing. **d** Orientation distribution of the particles used for 3D electron density reconstruction. **e** FSC curves of the local electron density map refined for the dimeric TLR3 unit. **f** The electron density map is colored according to the local resolution estimation. **g** Final electron density map after combining local refinement maps for each dimeric TLR3 unit. Two local refinement maps in (**c**) were combined using the Phenix program to generate the consensus map.

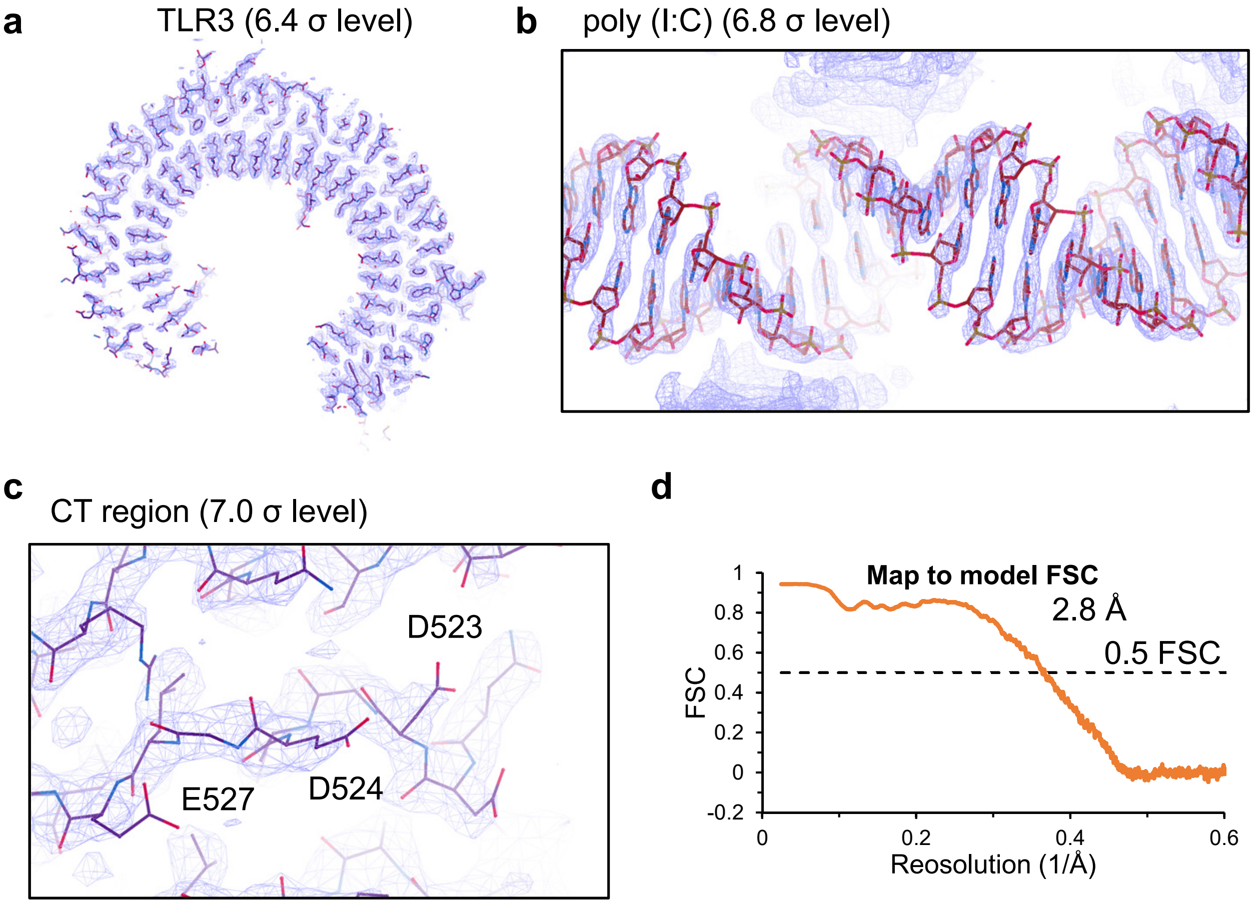

Supplementary Fig. 3 | Cryo-EM map of the TLR3-poly (I:C) complex in the clustered state. a Cryo-EM map superimposed with the refined model of TLR3. **b** Close-up view of the poly (I:C) region. **c** Close-up view of the CT region. **d** The map *vs.* model FSC curve.

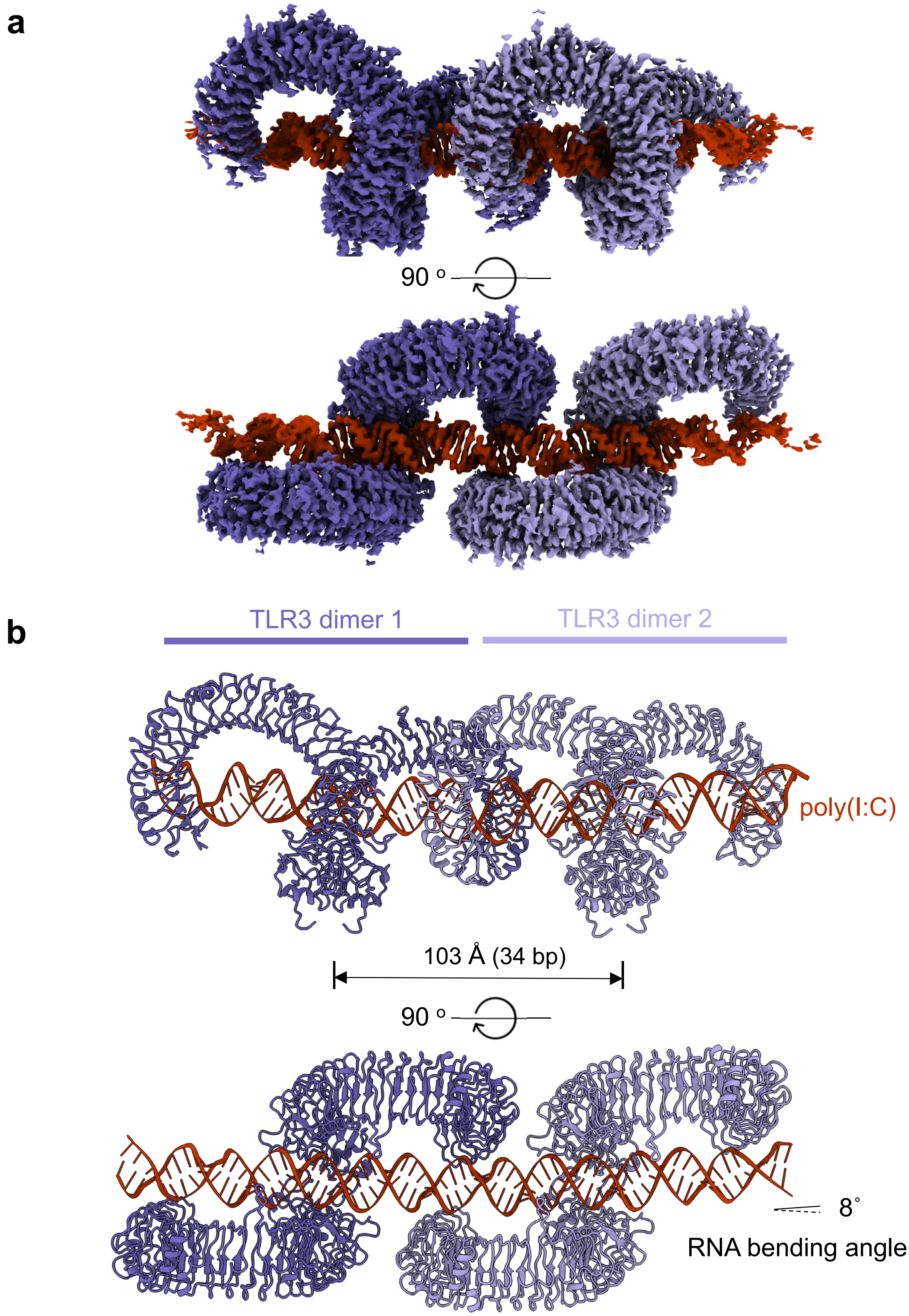

Supplementary Fig. 4 | Overall structure of TLR3-poly(I:C) complex. a Cryo-EM electron density map. **b** the atomic model of the TLR3-poly(I:C) complex. The TLR3 dimeric units are colored in purple and light purple, respectively. Poly(I:C) is colored in red. TLR3 dimers formed a cluster with a regular spacing of 103 Å or 34 bp of poly(I:C). The RNA double helix was bent by ~8 degrees.

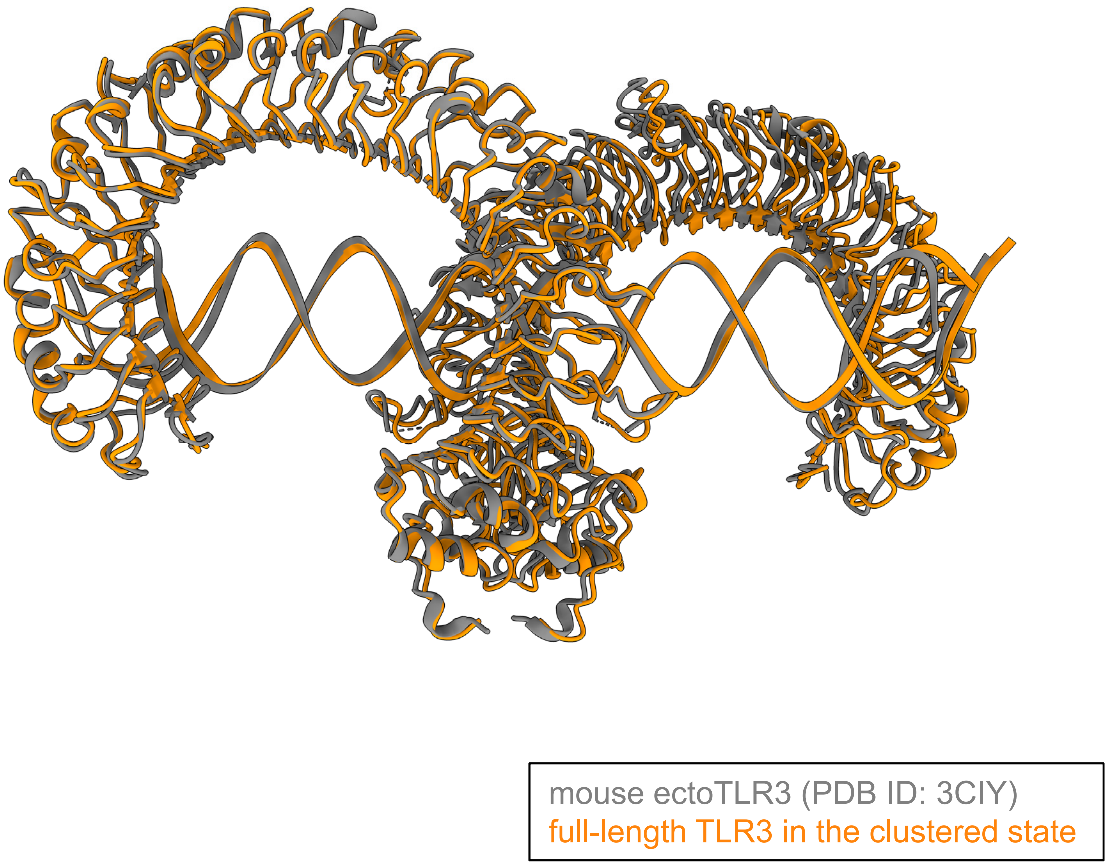

Supplementary Fig. 5 | Structural comparison of the human full-length TLR3 and the mouse ectoTLR3 (PDB ID: 3CIY).

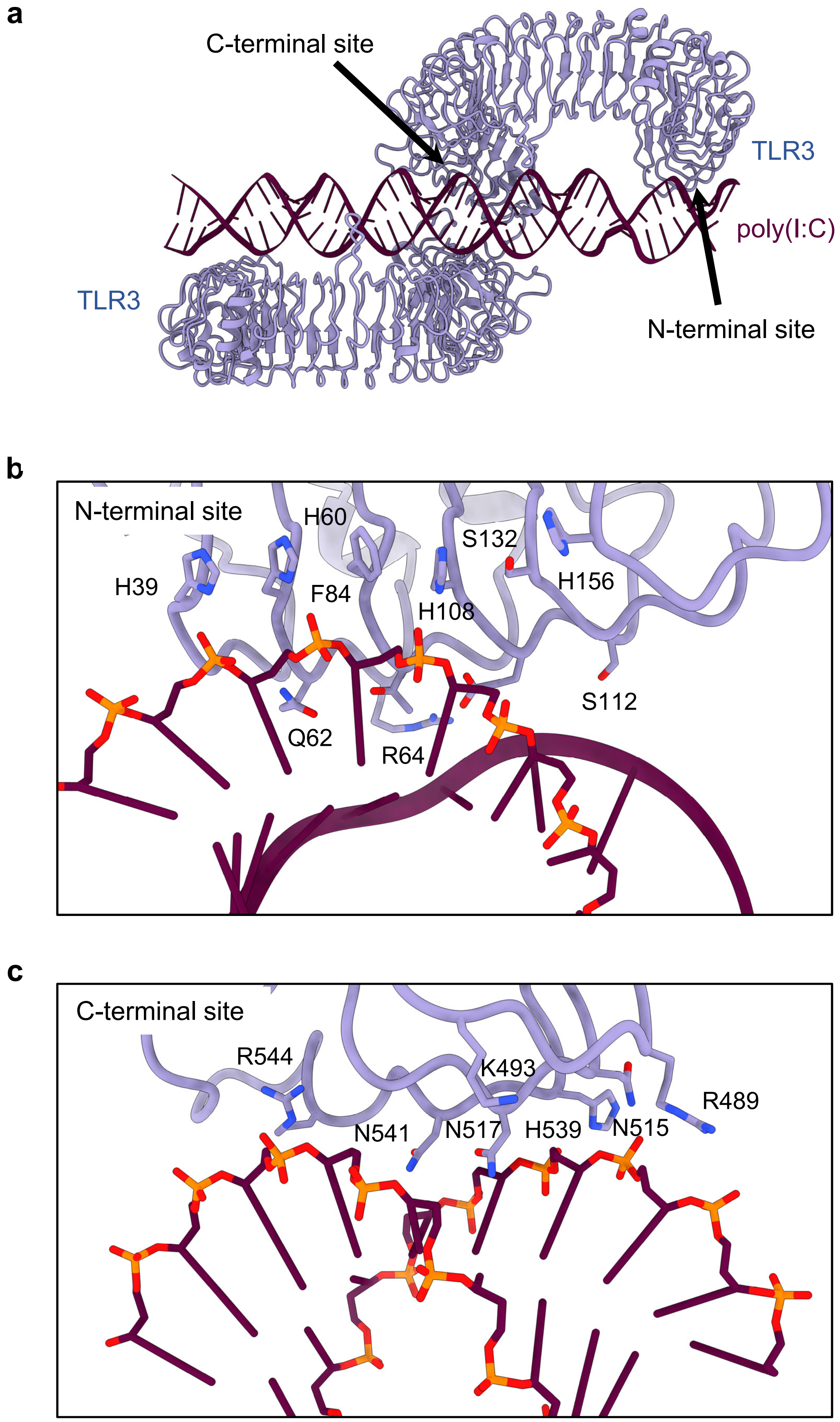

Supplementary Fig. 6 | The poly(I:C) binding sites of TLR3. a Overall structure and **b, c** Close-up view of the RNA binding sites. Residues involved in RNA binding are shown.

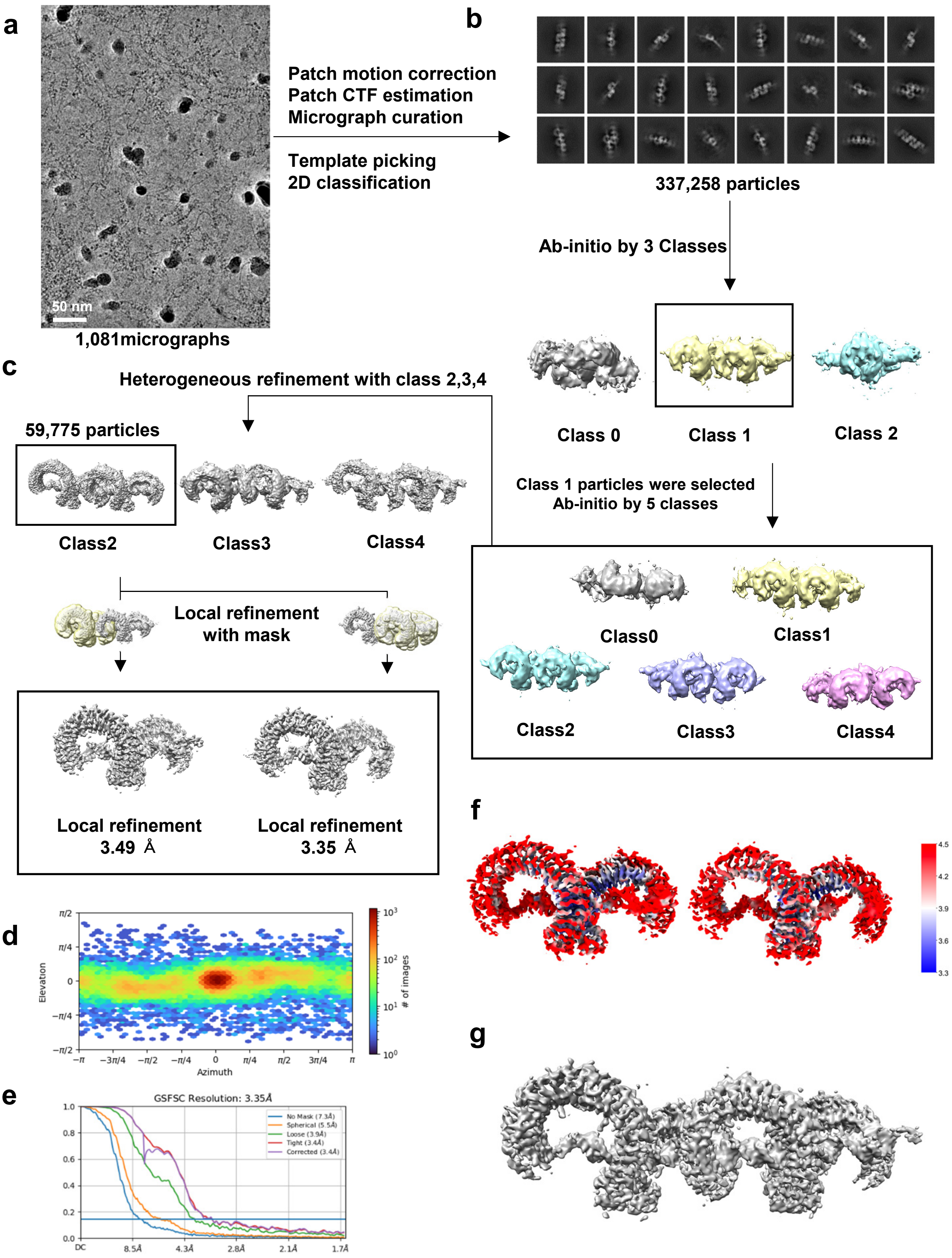
a
50 nm
1,081micrographs
Patch motion correction
Patch CTF estimation
Micrograph curation
Template picking
2D classification
b
337,258 particles
Ab-initio by 3 Classes
Class 0
Class 1
Class 2
Class 1 particles were selected
Ab-initio by 5 classes
Class0
Class1
Class2
Class3
Class4
c
Heterogeneous refinement with class 2,3,4
59,775 particles
Class2
Class3
Class4
Local refinement
with mask
Local refinement
3.49 Å
Local refinement
3.35 Å
d
Elevation
Azimuth
# of images
e
GSFSC Resolution: 3.35Å
No Mask (7.3Å)
Spherical (5.5Å)
Loose (3.9Å)
Tight (3.4Å)
Corrected (3.4Å)
f
g

Supplementary Fig. 7 | Cryo-EM data processing of the ectoTLR3-poly(I:C) complex in the clustered state. **a** Representative micrograph of the ectoTLR3-poly(I:C) complex. **b** Selected 2D class average images of the ectoTLR3-poly(I:C) complex. **c** Summary of cryo-EM data processing. **d** Orientation distribution of the particles used for 3D electron density reconstruction. **e** FSC curves of the local electron density map refined for the dimeric TLR3 unit. **f** The electron density map is colored according to the local resolution estimation. **g** Final electron density map after combining local refinement maps for each dimeric TLR3 unit. Two local refinement maps in (**c**) were combined using the Phenix program to generate the consensus map.

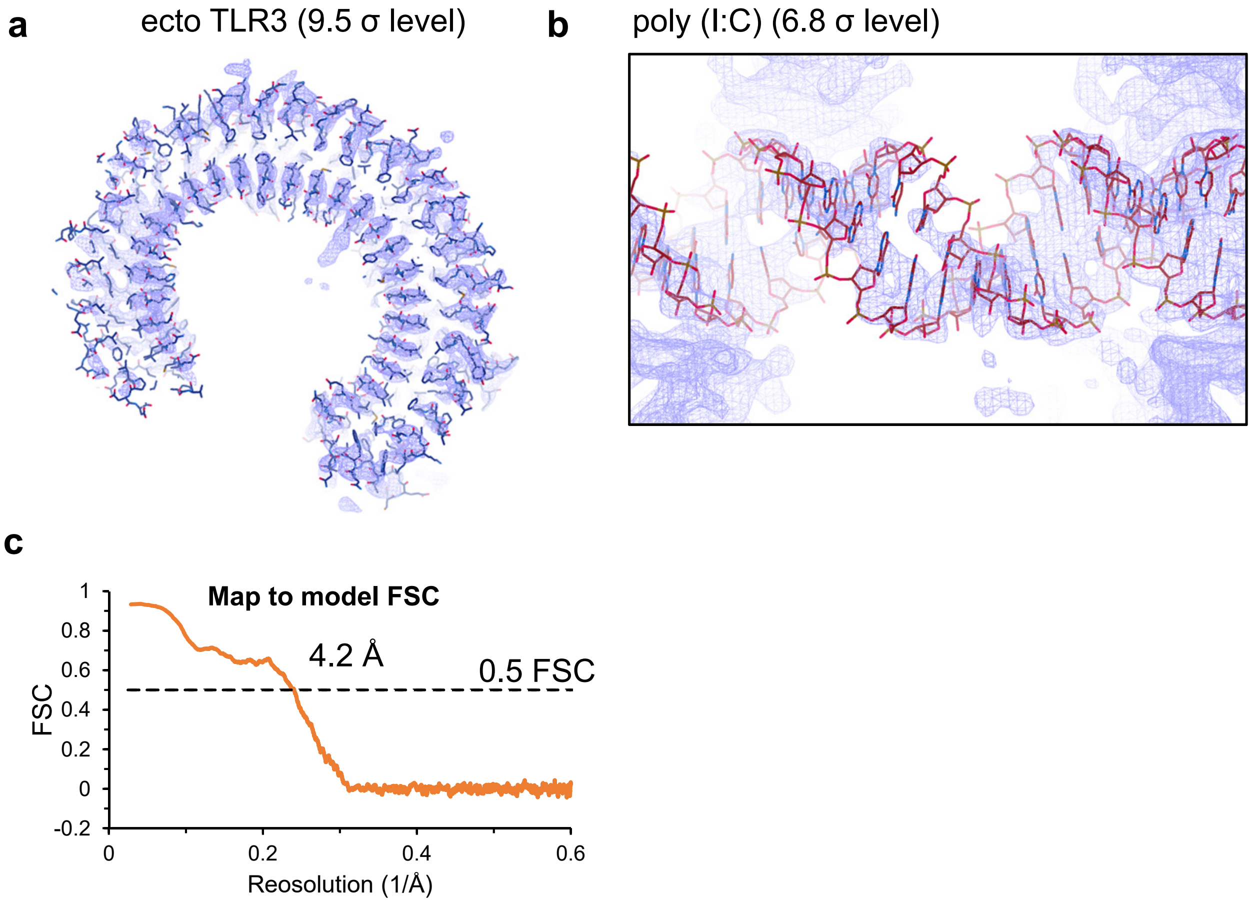

Supplementary Fig. 8 | Cryo-EM map of the ectoTLR3-poly (I:C) complex in the clustered state. a Cryo-EM map superimposed with the refined model of ectoTLR3. **b** Close-up view of the poly (I:C) region. **c** The map *vs.* model FSC curve.

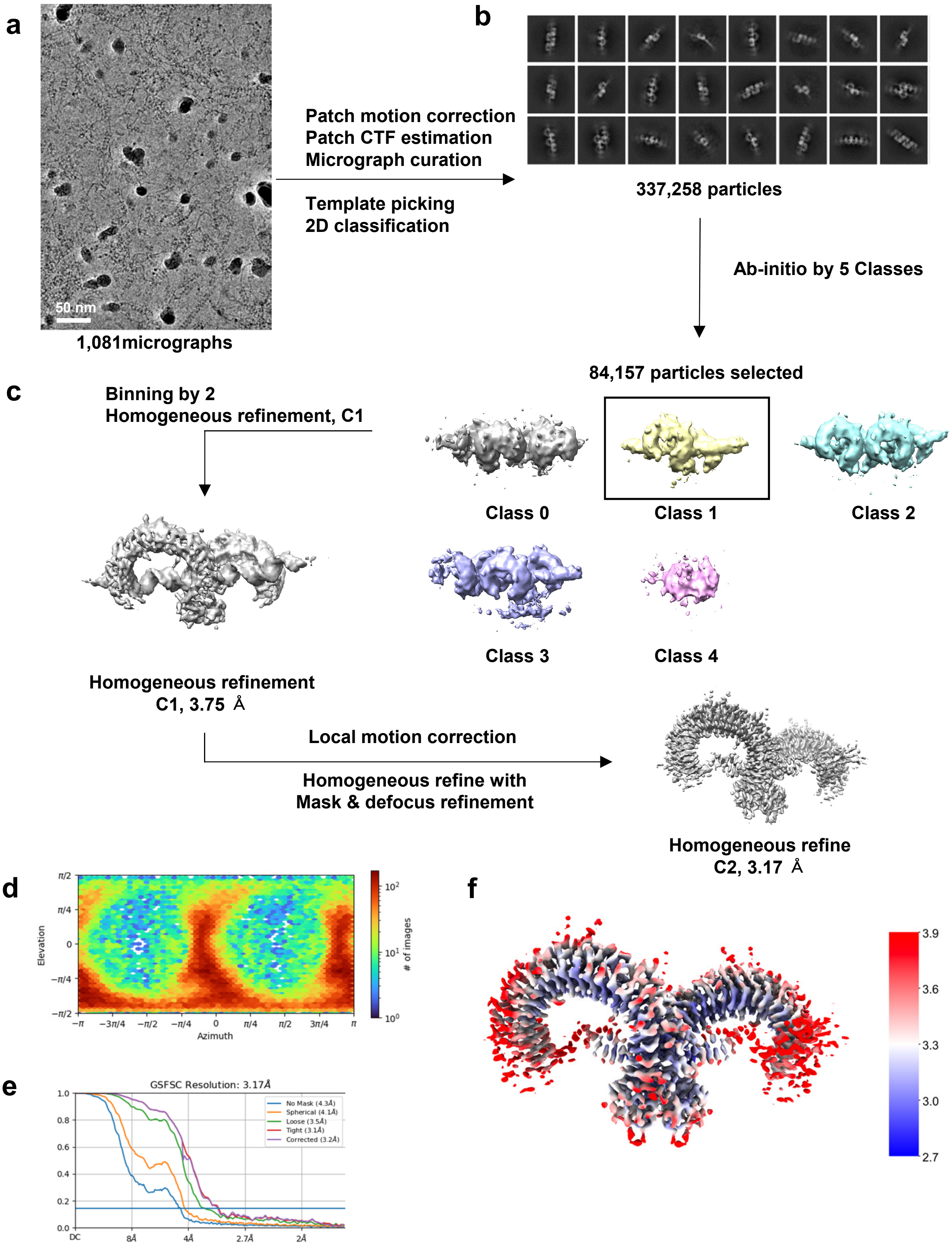

Supplementary Fig. 9 | Cryo-EM data processing of the ectoTLR3-poly(I:C) complex in the dimeric state. a Representative micrograph of the ectoTLR3-poly(I:C) complex. **b** Selected 2D class average images of the ectoTLR3-poly(I:C) complex. **c** Summary of cryo-EM data processing. **d** Orientation distribution of the particles used for 3D electron density reconstruction. **e** FSC curves of the refinement map. **f** Local resolution distribution of the refined map.

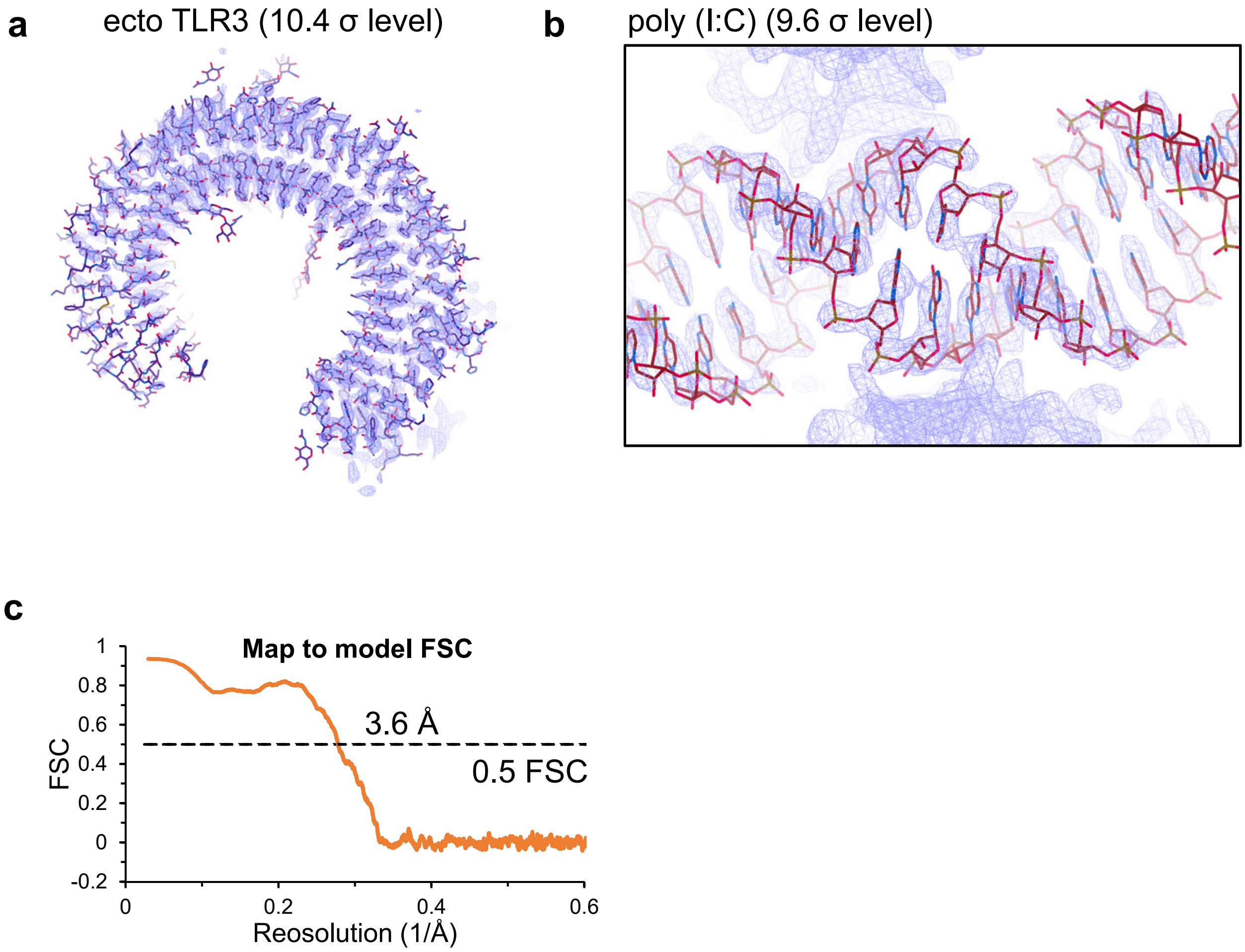

Supplementary Fig. 10 | Cryo-EM map of the ectoTLR3-poly (I:C) complex in the dimeric state. a Cryo-EM map superimposed with the refined model of ectoTLR3. **b** Close-up view of the poly (I:C) region. **c** The map *vs.* model FSC curve.

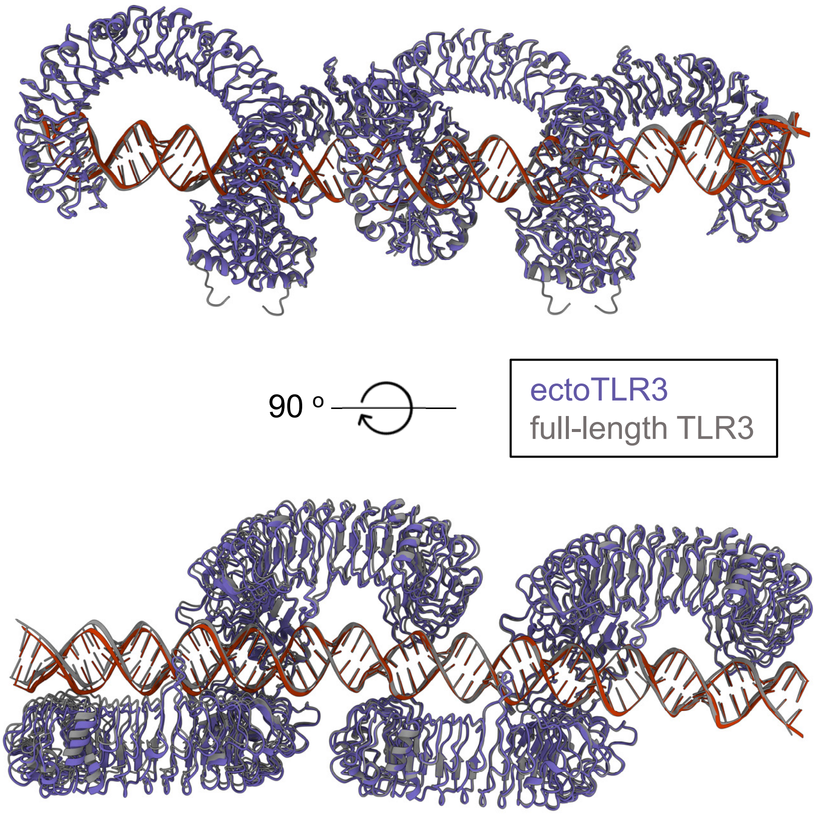

Supplementary Fig. 11 | Structural comparison of the ectoTLR3 and the full-length TLR3 in complex with poly(I:C).

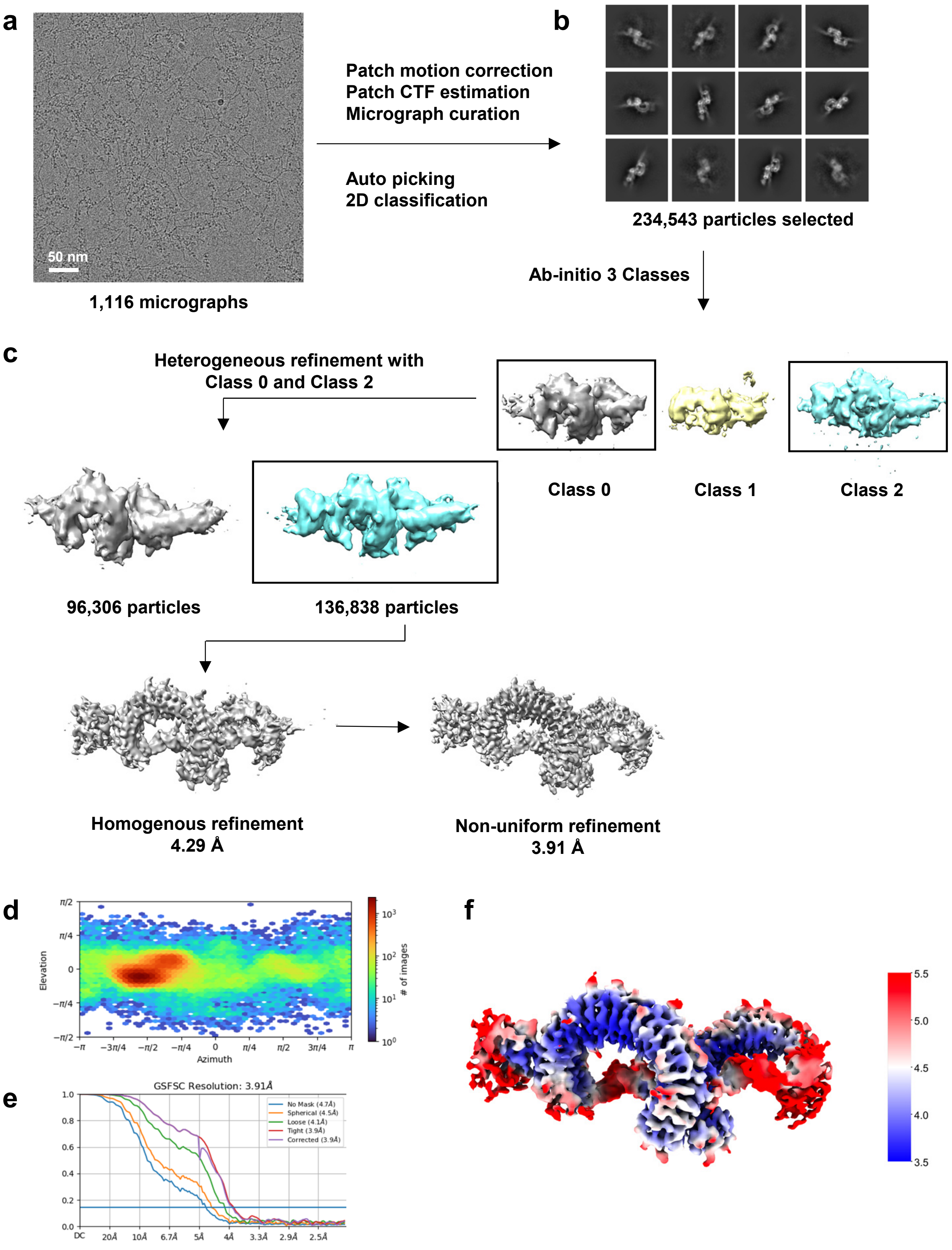

Supplementary Fig. 12 | Cryo-EM data processing of the ectoTLR3-mAb12-poly(I:C) complex. An scFv form of the mAb12 antibody was used for complex formation. **a, b** Representative micrograph (**a**) and 2D class average images (**b**) of the ectoTLR3-Fab12-poly(I:C) complex. **c** Summary of cryo-EM data processing. **d** Orientation distribution, **e** FSC curves, and **f** local resolution distribution of the refined map.

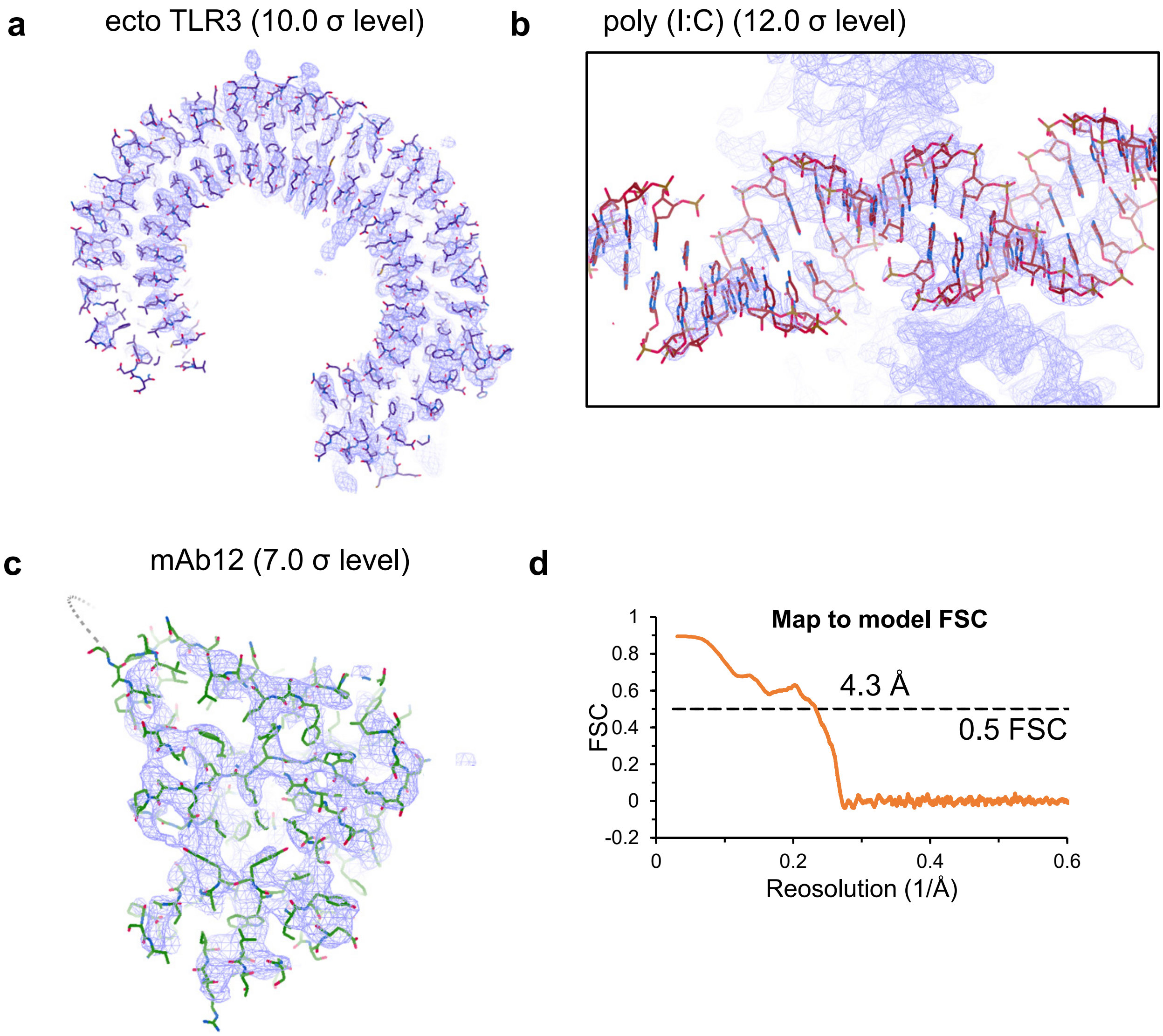

Supplementary Fig. 13 | Cryo-EM map of the ectoTLR3-mAb12-poly (I:C) complex. **a** Cryo-EM map superimposed with the refined model of ectoTLR3. **b** Close-up view of the poly (I:C) region. **c** Close-up view of the mAb12 region. **d** The map *vs.* model FSC curve.

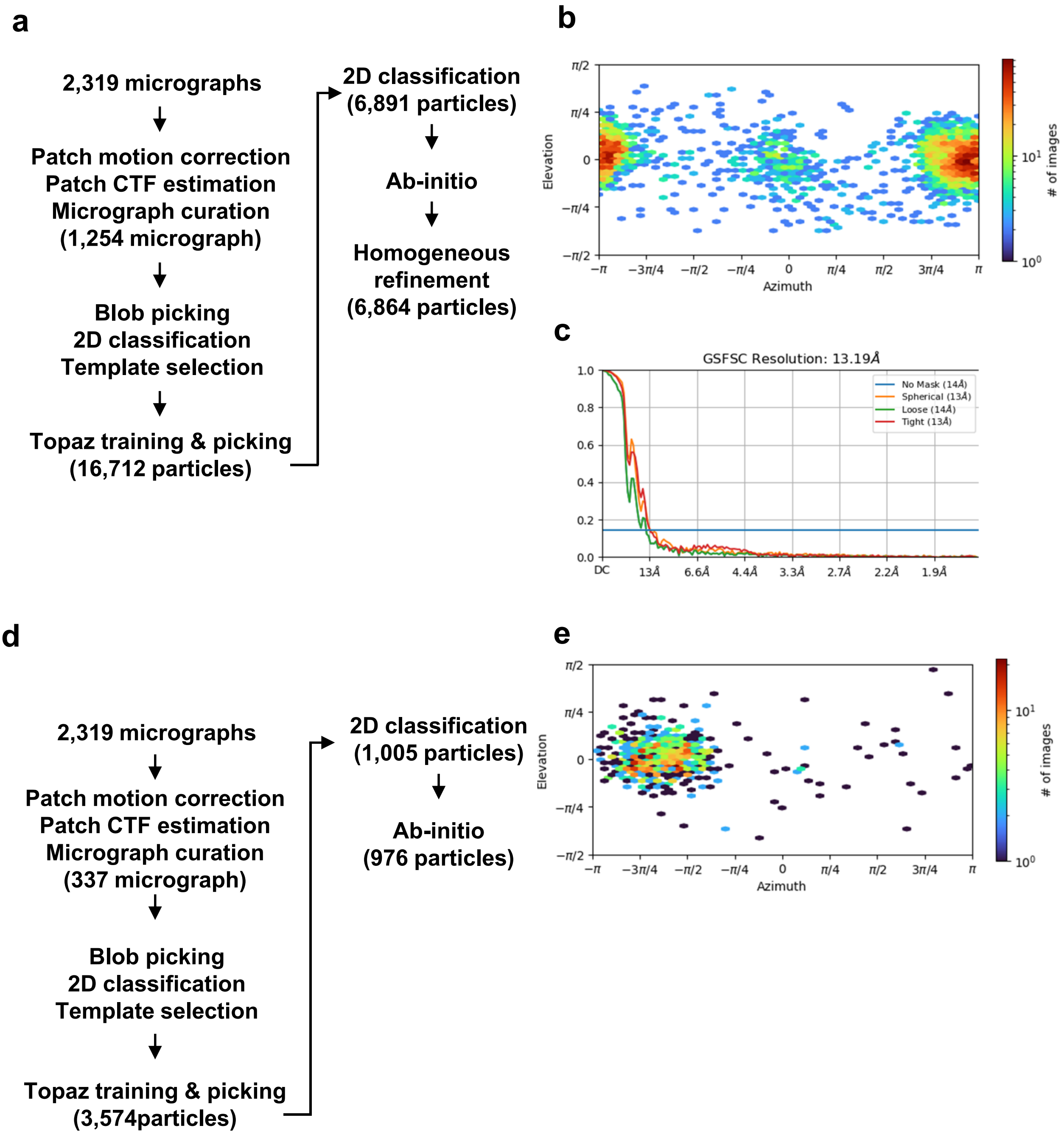

Supplementary Fig. 14 | Cryo-EM data processing of the TLR3-mAb12-poly (I:C) complex. a Summary of cryo-EM data processing for TLR3 particles with bound mAb12. **b** Orientation distribution of the particles. **c** FSC curve of TLR3 particles with bound mAb12. **d** Summary of cryo-EM data processing of TLR3 particles without bound mAb12. **e** Orientation distribution of TLR3 particles without bound mAb12.

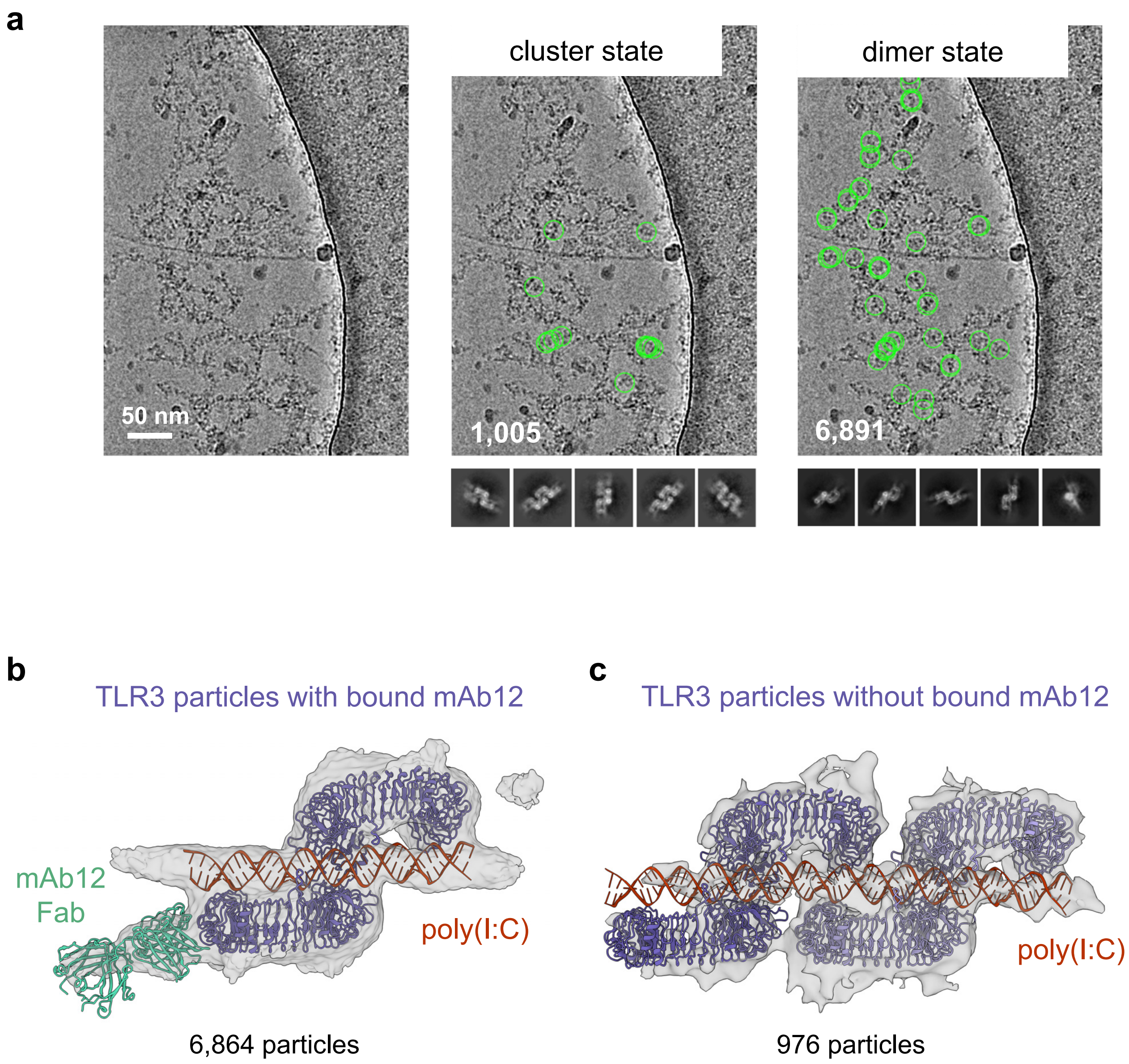

Supplementary Fig. 15 | Inhibition of full-length TLR3 clustering by a blocking antibody. a Representative micrograph (n=2,319) of the TLR3-mAb12-poly(I:C) complex. The antibody blocked cluster formation. An Fab form of the antibody mAb12 was used for complex formation. TLR3 particles in the clustered (middle) and dimeric states (right) are marked with green circles. 2D class averaged images of TLR3 are shown below. The molar ratio of mAb12 vs TLR3 was 0.5:1. **b** Atomic model of the TLR3-mAb12-poly(I:C) complex. The TLR3-mAb12-poly(I:C) model is fitted to the cryo-EM density map using the Chimera program. The structure of the CH1 domain of the Fab is obtained from the crystallographic structure of the mAb12-ectoTLR3 complex (PDB id: 3ULU). **c** TLR3 particles in the clustered state did not have bound antibodies.

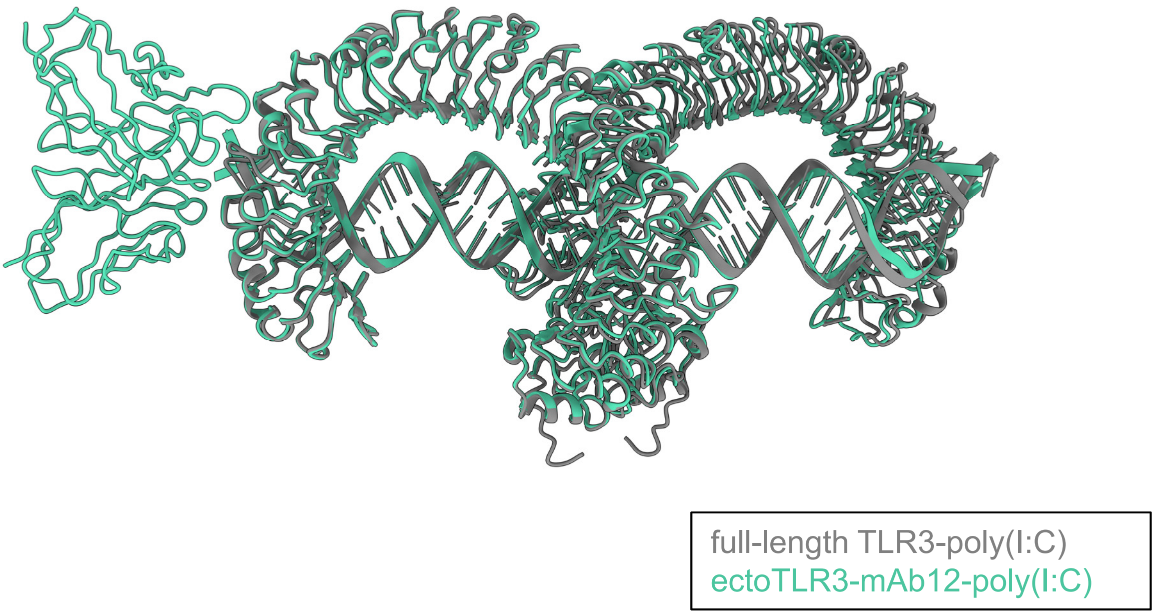

Supplementary Fig. 16 | Structural comparison of TLR3 with and without the bound antibody. The Cα r.m.s.d. of the two structures is 0.675 Å.

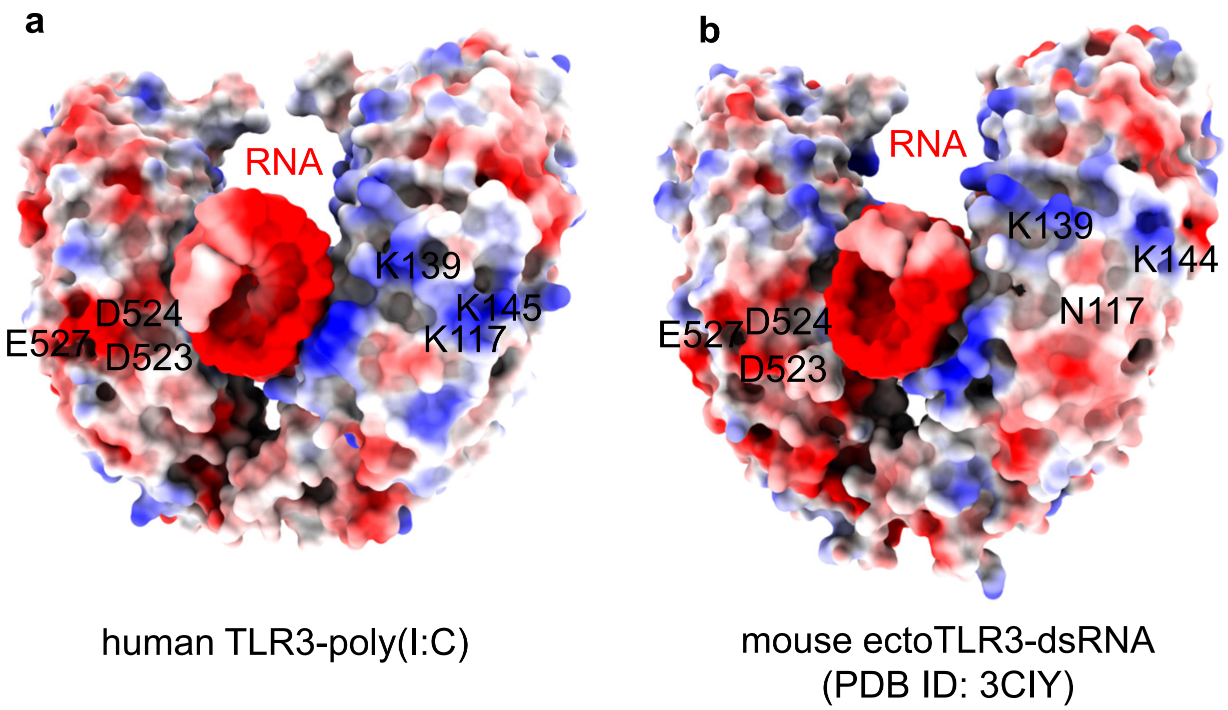

Supplementary Fig. 17 | Surface charge distribution of the interface between two dimeric TLR3 units in the cluster. a, b The surface charge distribution of the human TLR3-dsRNA complex (**a**), and the mouse ectoTLR3-dsRNA complex (**b**). The negatively charged and positively charged surfaces are colored in red and blue, respectively. The residues involved in the cluster formation is written.

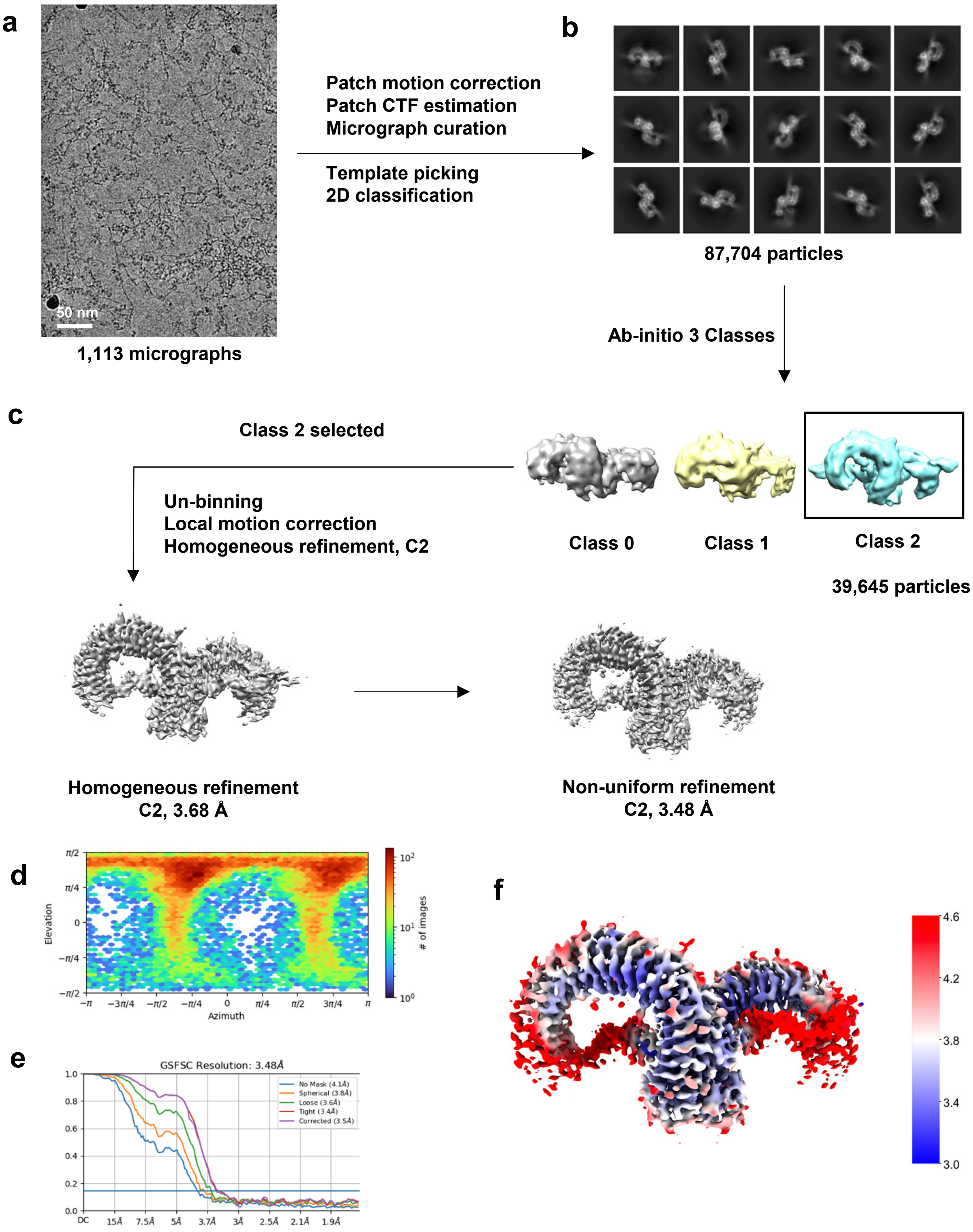

Supplementary Fig. 18 | Cryo-EM data processing of the NT-mut ectoTLR3-poly(I:C) complex in the dimeric state. a, b Representative micrograph (**a**) and 2D class average images (**b**) of the NT-mut ectoTLR3-poly(I:C) complex. **c** Summary of cryo-EM data processing. **d** Orientation distribution, **e** FSC curves, and **f** local resolution distribution of the refined map.

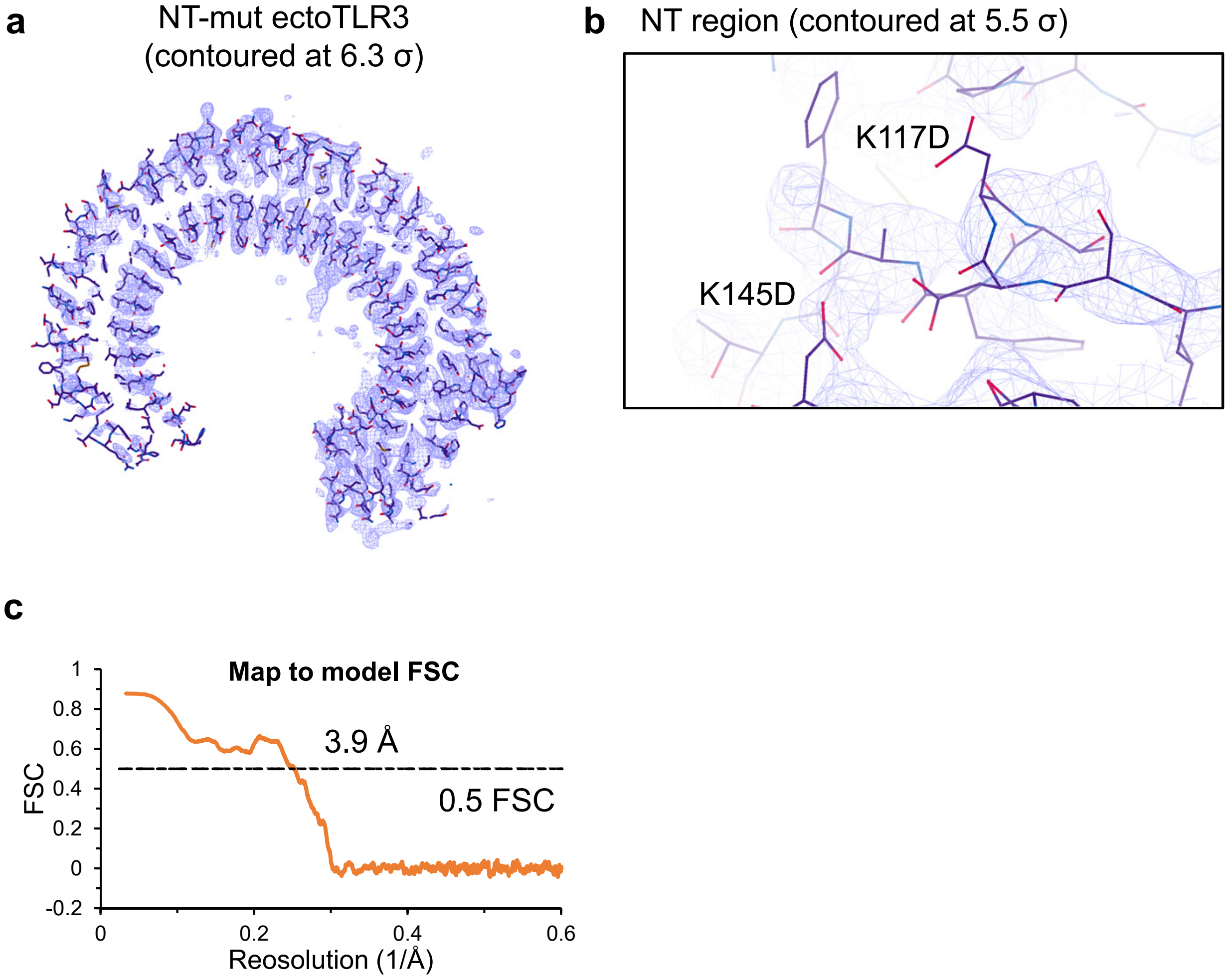

Supplementary Fig. 19 Cryo-EM map of the NT-mut ectoTLR3. a Cryo-EM map superimposed with the refined model of NT-mut ectoTLR3. **b** Close-up view of the NT region of NT-mut ectoTLR3. **c** The map *vs.* model FSC curve.

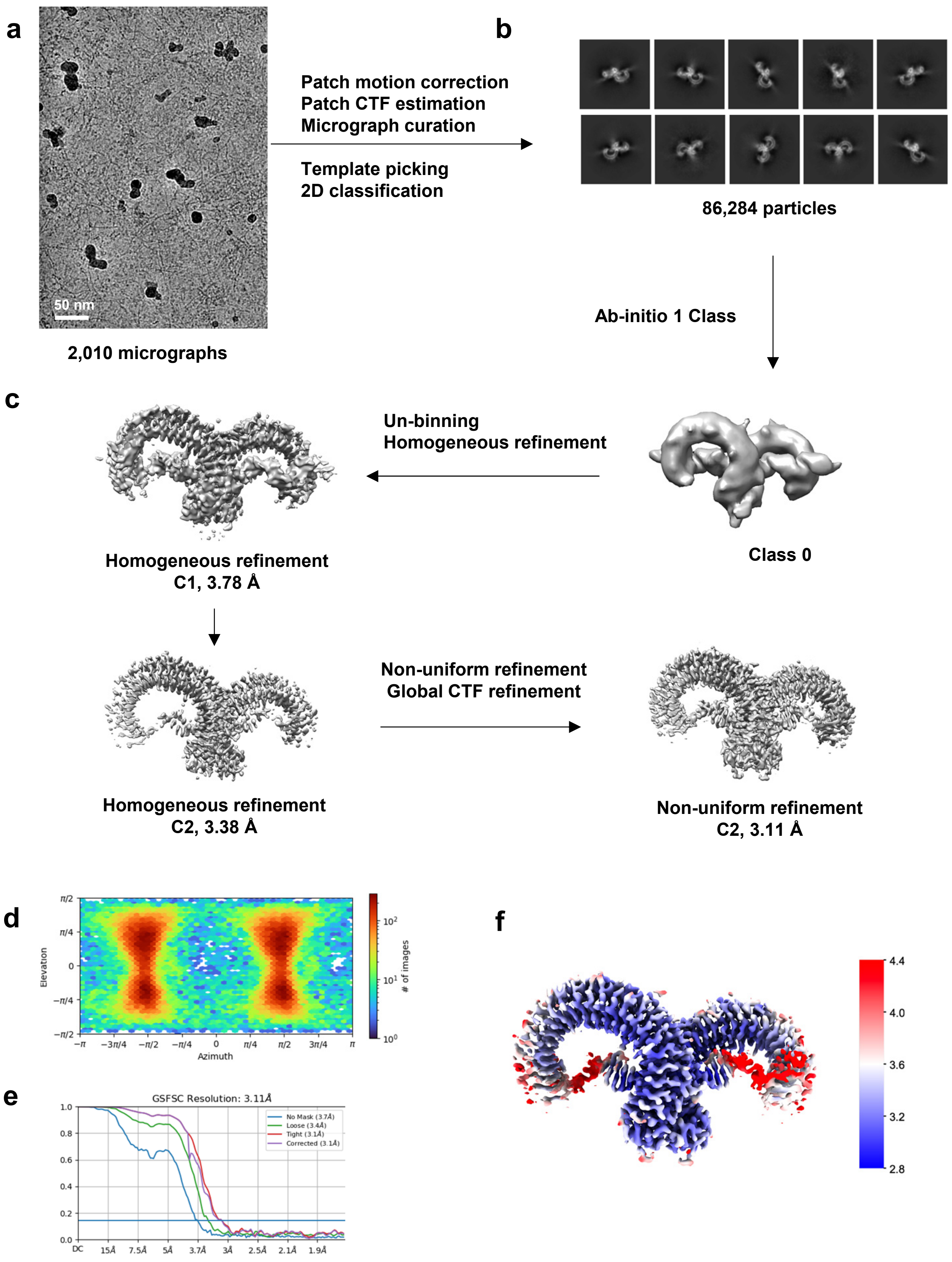

Supplementary Fig. 20 | Cryo-EM data processing of the CT-mut ectoTLR3-poly(I:C) complex in the dimeric state. a, b Representative micrograph (**a**) and 2D class average images (**b**) of the CT-mut ectoTLR3-poly(I:C) complex. **c** Summary of cryo-EM data processing. **d** Orientation distribution, **e** FSC curves, and **f** local resolution distribution of the refined map.

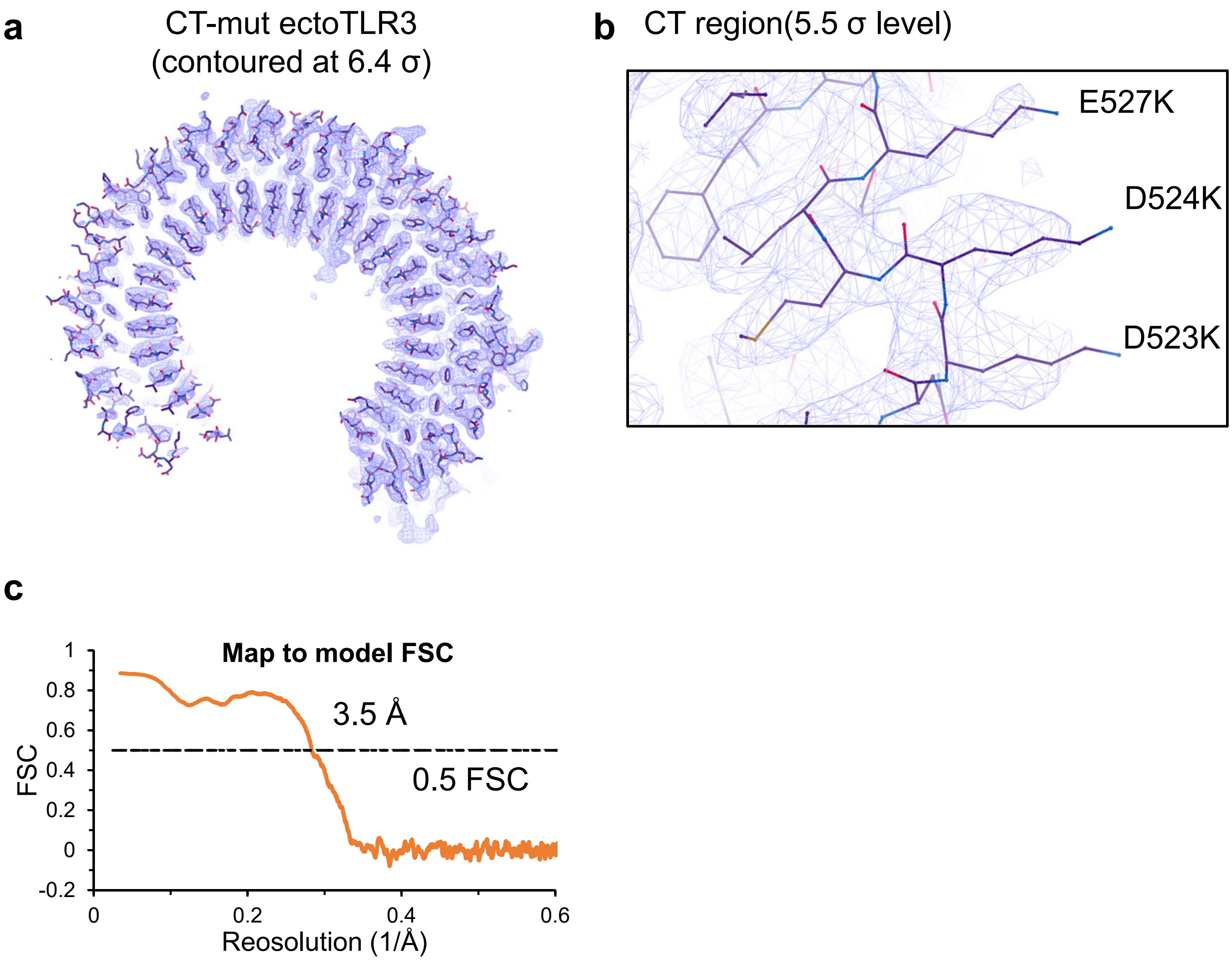

Supplementary Fig. 21 | Cryo-EM map of the CT-mut ectoTLR3. a Cryo-EM map superimposed with the refined model of CT-mut ectoTLR3. **b** Close-up view of the CT region of CT-mut ectoTLR3. **c** The map *vs.* model FSC curve.

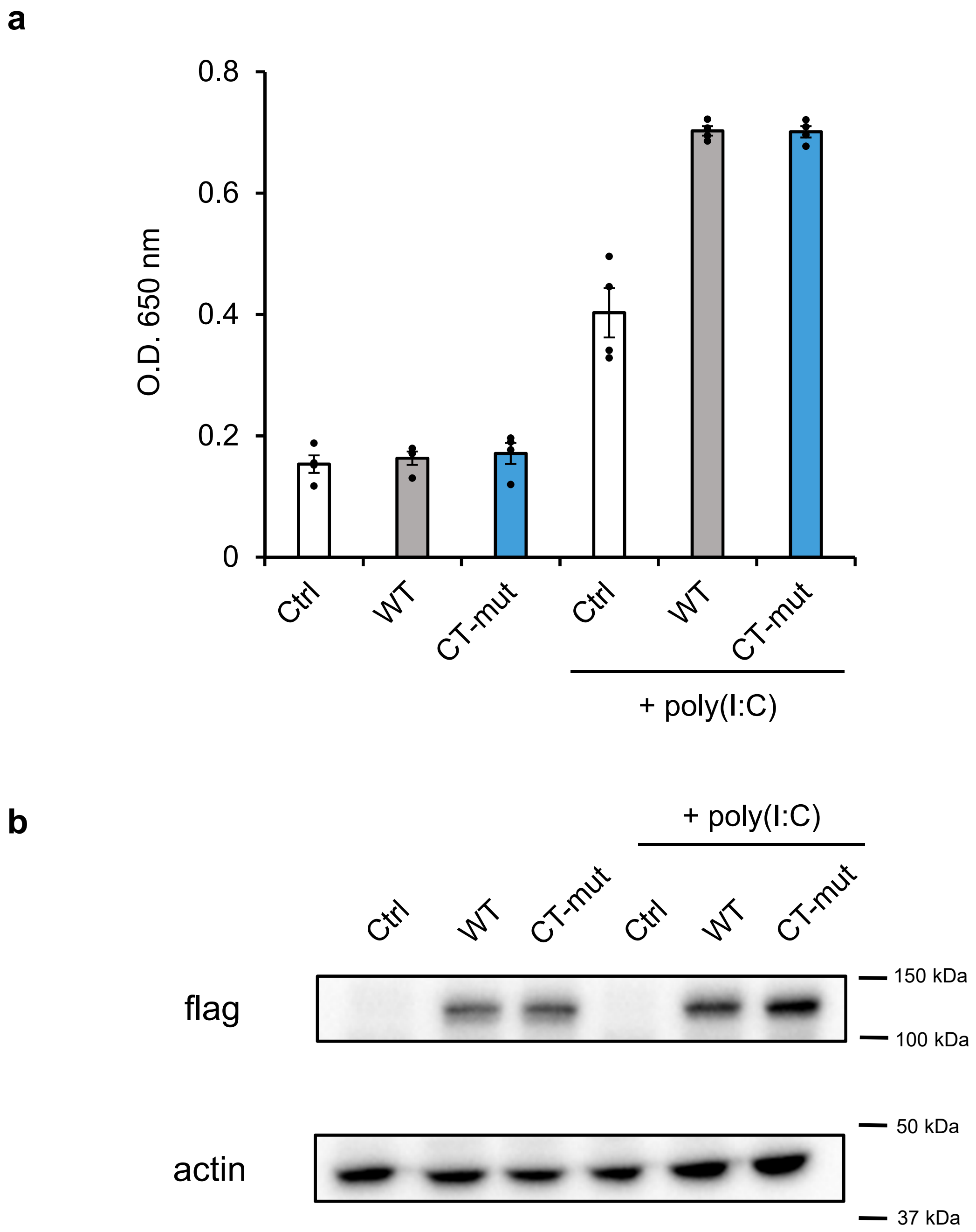

Supplementary Fig. 22 | Effect of a charge-reversion mutation on TLR3 signaling activity. a TLR3-dependent NF-κB assay. Transfected HEK-Blue Null cells were stimulated with poly(I:C). Reporter activities of the cells transfected with null (Ctrl), wild-type, CT-mut TLR3s are shown. N=4 independent experiments were performed. Data are presented as mean values +/- SEM. **b** Western blot analysis of the transfected cells with anti-FLAG antibody. Representative blot images from the four independent experiment are shown. Source data are provided as a Source Data file.

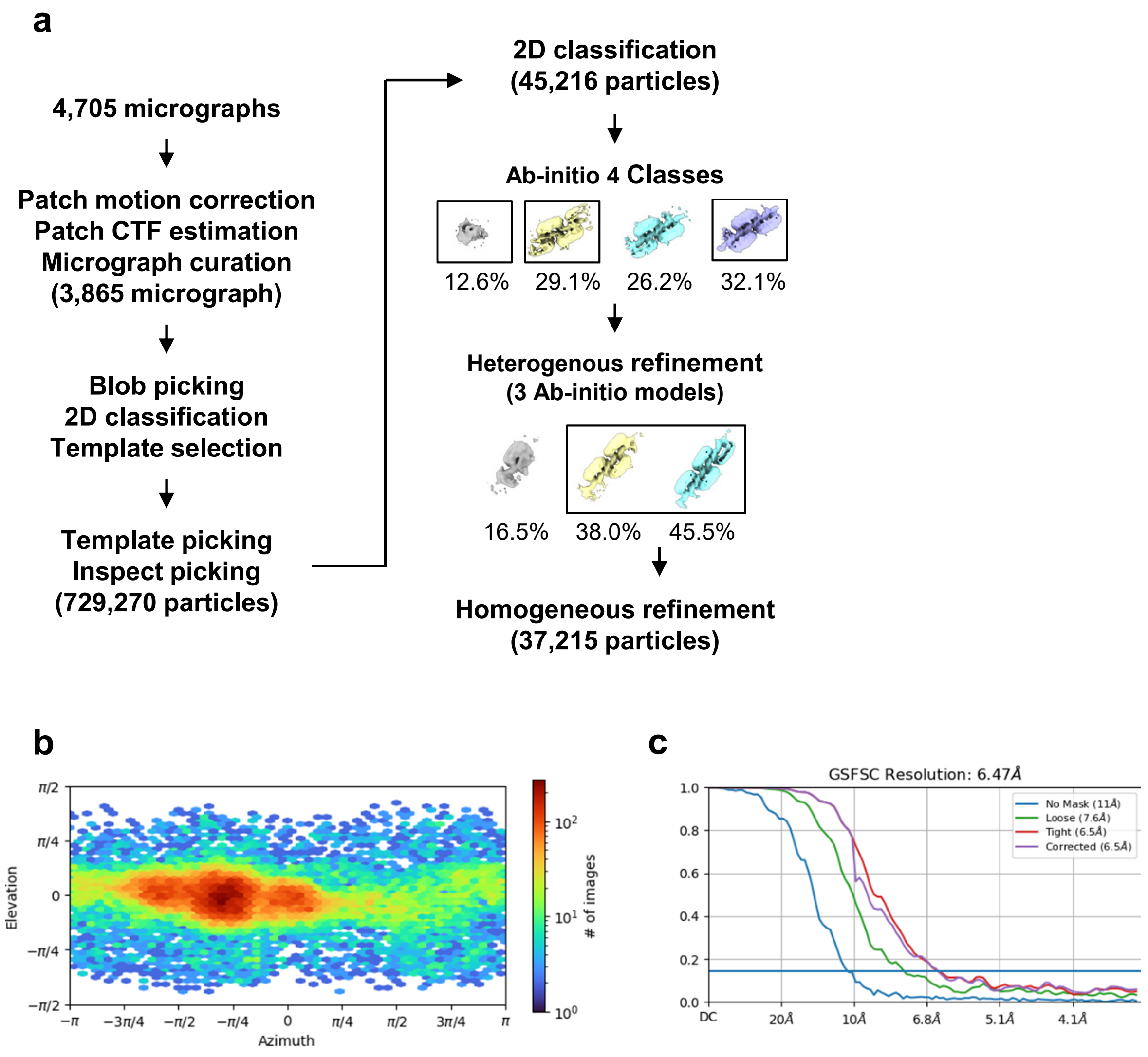

Supplementary Fig. 23 | Cryo-EM data processing of the TLR3(A795H)-poly(I:C) complex. a Summary of cryo-EM data processing. **b** Orientation distribution of the particles. **c** FSC curves.

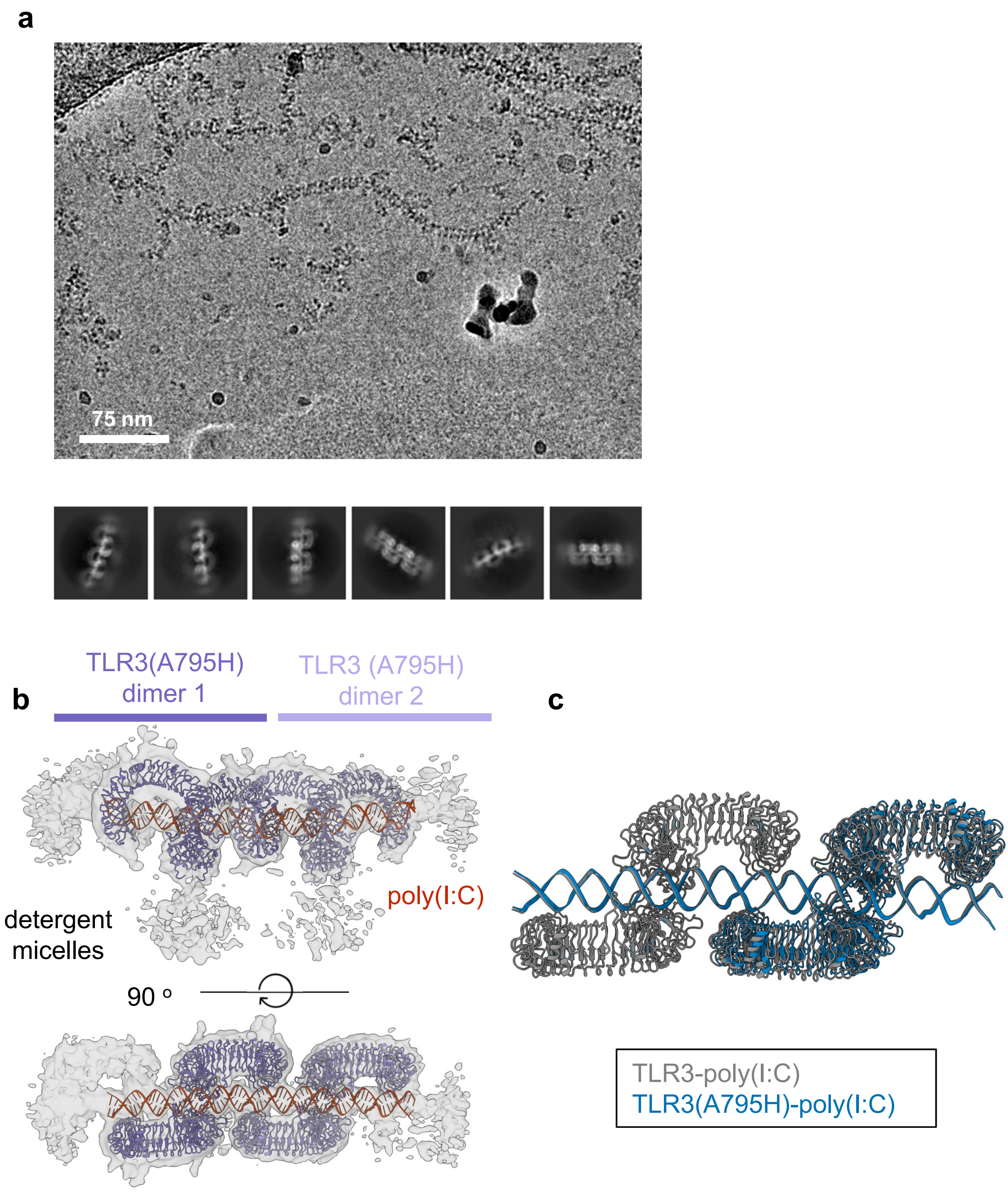

Supplementary Fig. 24 | Effect of BB loop mutation on the cluster formation of TLR3. a Representative micrograph (n=4,705) of the TLR3(A795H)-poly(I:C) complex. 2D class averaged images of TLR3 are shown below. **b** Atomic model of the TLR3(A795H)-poly(I:C) complex. The refined models of the dimeric TLR3-poly(I:C) complex are fitted to the cryo-EM density map using the Chimera and Phenix programs. **c** Structural comparison of wild-type and the A795H mutant of TLR3. The two structures are superimposed using one of the dimeric units as the reference.

Forward : GAAAGGGACTTTGAGcatGGTGTTTTTGAA

Reverse : TAGTTCAAAAACACCatgCTCAAAGTCCCT

Supplementary Fig. 25 | Primers for the A795H mutagenesis experiment.

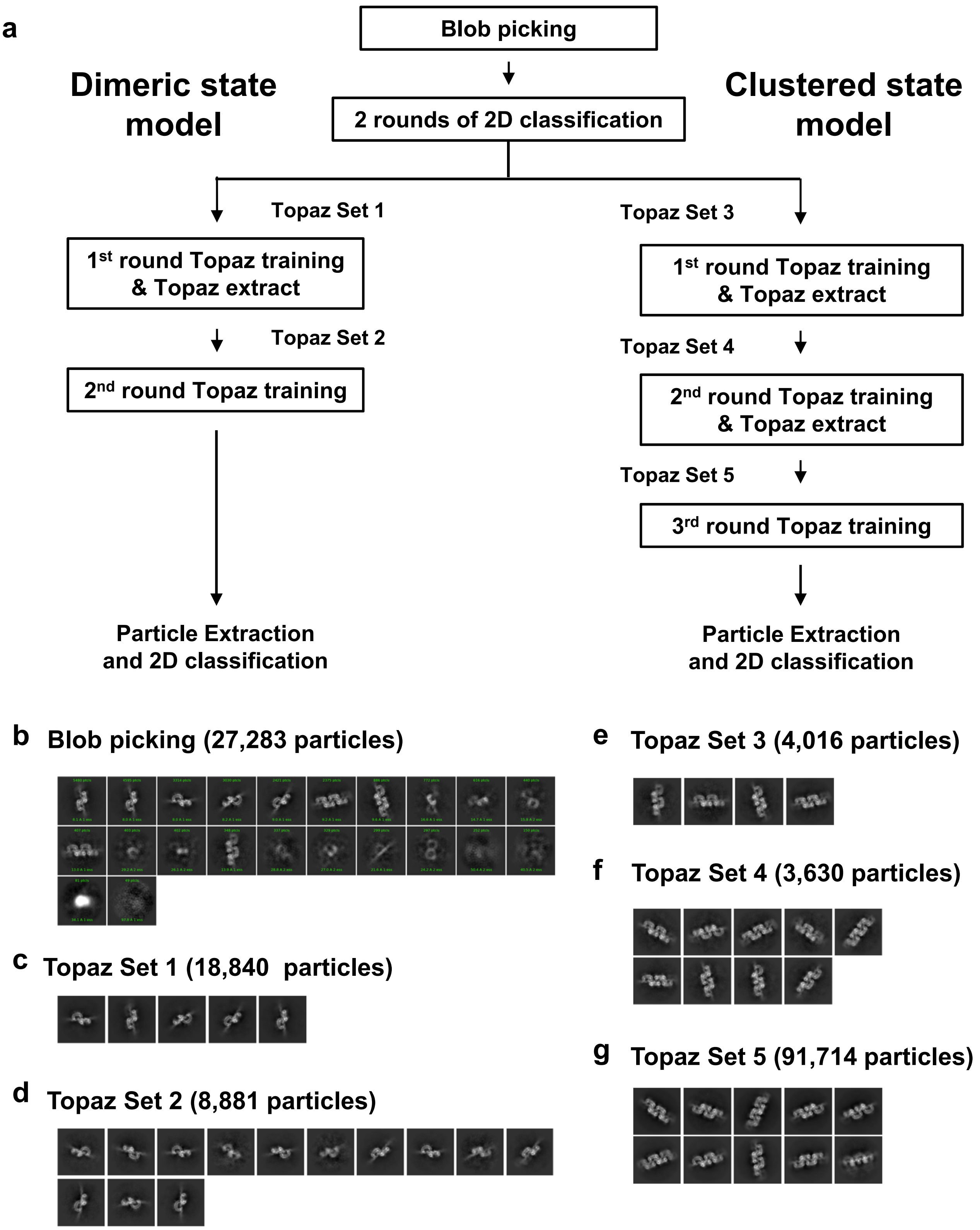

Supplementary Fig. 26 | Topaz training and particle-picking workflow. a Summary of the Topaz training and particle-picking method. **b** 2D class averaged images of particles picked by the blob-picking method. Particle images from the ectoTLR3-poly (I:C) data are shown as the representative examples. **c-g** 2D class averaged images of input particles for each Topaz training step.

a

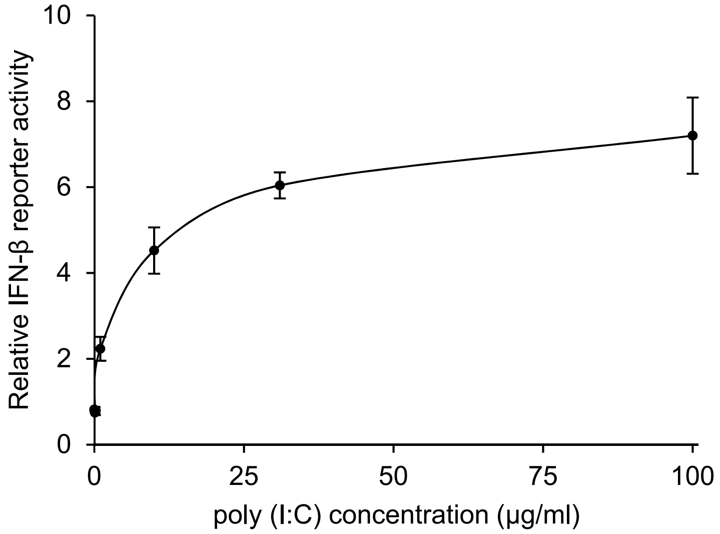

b

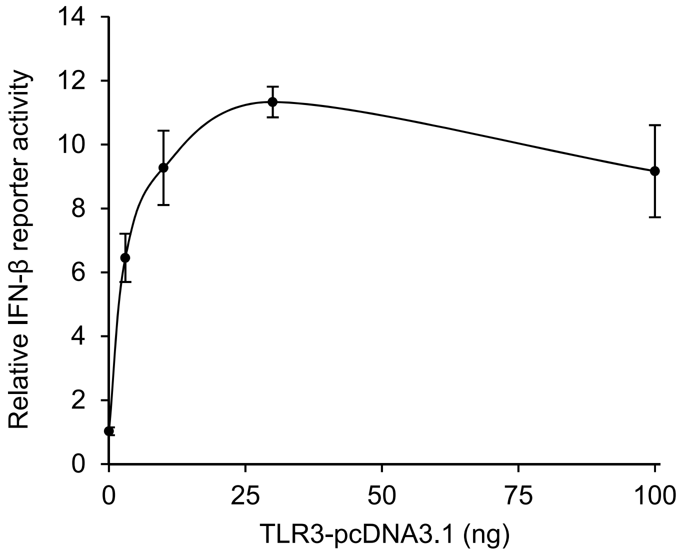

Supplementary Fig. 27 | TLR3-dependent activation of the INF-β promoter by poly(I:C) treatment. a After transfection with TLR3-pcDNA3.1 and IFN-Beta_pGL3, IFN-β reporter activity was measured at varying poly(I:C) concentrations. **b** The reporter activity was also monitored by changing TLR3-pcDNA3.1 concentrations. The result did not show cooperativity in TLR3 signaling. N=3 independent experiments were performed. Data are presented as mean values +/- SEM. Source data are provided as a Source Data file.
