# Supplementary material for: TLR3 forms a highly organized cluster when bound to a poly(I:C) RNA ligand
Source: Nat Commun. 2022 Nov 12;13:6876. doi: 10.1038/s41467-022-34602-0 (PMC9653405; doi:10.1038/s41467-022-34602-0)
Supplement: Supplementary file 2 — Reporting Summary [file 41467_2022_34602_MOESM2_ESM.pdf]

nature portfolio

Corresponding author(s): Ji Won Kim, Jie-Oh Lee

Last updated by author(s): Oct. 18th, 2022

Reporting Summary

Nature Portfolio wishes to improve the reproducibility of the work that we publish. This form provides structure for consistency and transparency in reporting. For further information on Nature Portfolio policies, see our Editorial Policies and the Editorial Policy Checklist.

Statistics

For all statistical analyses, confirm that the following items are present in the figure legend, table legend, main text, or Methods section.

n/a	Confirmed	
☐	☒	The exact sample size (*n*) for each experimental group/condition, given as a discrete number and unit of measurement
☐	☒	A statement on whether measurements were taken from distinct samples or whether the same sample was measured repeatedly
☐	☒	The statistical test(s) used AND whether they are one- or two-sided<br>*Only common tests should be described solely by name; describe more complex techniques in the Methods section.*
☐	☒	A description of all covariates tested
☒	☐	A description of any assumptions or corrections, such as tests of normality and adjustment for multiple comparisons
☐	☒	A full description of the statistical parameters including central tendency (e.g. means) or other basic estimates (e.g. regression coefficient) AND variation (e.g. standard deviation) or associated estimates of uncertainty (e.g. confidence intervals)
☐	☒	For null hypothesis testing, the test statistic (e.g. *F*, *t*, *r*) with confidence intervals, effect sizes, degrees of freedom and *P* value noted<br>*Give P values as exact values whenever suitable.*
☒	☐	For Bayesian analysis, information on the choice of priors and Markov chain Monte Carlo settings
☒	☐	For hierarchical and complex designs, identification of the appropriate level for tests and full reporting of outcomes
☒	☐	Estimates of effect sizes (e.g. Cohen's *d*, Pearson's *r*), indicating how they were calculated

Our web collection on statistics for biologists contains articles on many of the points above.

Software and code

Policy information about availability of computer code

Data collection	EPU 2.13.0.3175REL
Data analysis	UCSF Chimera (v 1.15), UCSF ChimeraX (v 1.2.5), cryoSPARC(v 3.2), Topaz(v 0.2.3), COOT (v 0.9.6), Phenix (v 1.19.2), GraphPad Prism 9

For manuscripts utilizing custom algorithms or software that are central to the research but not yet described in published literature, software must be made available to editors and reviewers. We strongly encourage code deposition in a community repository (e.g. GitHub). See the Nature Portfolio guidelines for submitting code & software for further information.

Data

Policy information about availability of data

All manuscripts must include a data availability statement. This statement should provide the following information, where applicable:

- Accession codes, unique identifiers, or web links for publicly available datasets
- A description of any restrictions on data availability
- For clinical datasets or third party data, please ensure that the statement adheres to our policy

The cryo-EM electron density maps and the atomic models were deposited Ito the Electron Microscopy Data Bank (EMDB) and the Protein Data Bank (PDB), respectively. Accession codes are 7WV3 [https://doi.org/10.2210/pdb7WV3/pdb] and EMD-32844 [https://www.ebi.ac.uk/emdb/entry/EMD-32844] for TLR3-poly(I:C) cluster data, 7WV4 [https://doi.org/10.2210/pdb7WV4/pdb] and EMD-32845 [https://www.ebi.ac.uk/emdb/entry/EMD-32845] for ectoTLR3-poly(I:C) cluster, 7WV5 [https://doi.org/10.2210/pdb7WV5/pdb] and EMD-32846 [https://www.ebi.ac.uk/emdb/entry/EMD-32846] for ectoTLR3-poly(I:C) dimeric, 7WVF

[https://doi.org/10.2210/pdb7WVF/pdb] and EMD-32852 [https://www.ebi.ac.uk/emdb/entry/EMD-32852] for ectoTLR3-mAb12-poly(I:C), 7WVJ [https://doi.org/10.2210/pdb7WVJ/pdb] and EMD-32853 [https://www.ebi.ac.uk/emdb/entry/EMD-32853] for NT-mut ectoTLR3-poly(I:C), 7WVE [https://doi.org/10.2210/pdb7WVE/pdb] and EMD-32851 [https://www.ebi.ac.uk/emdb/entry/EMD-32851] for CT-mut ectoTLR3-poly(I:C), respectively.
The electron density map of TLR3(A795H)-poly(I:C) clustered and TLR3-mAb12-poly(I:C) were deposited EMDB under accession code EMD-34361 [https://www.ebi.ac.uk/emdb/entry/EMD-34361] and EMD-34367 [https://www.ebi.ac.uk/emdb/entry/EMD-34367], respectively.
All raw data of reporter assay, statistic data, blot and gel images are available in Source data file.

Human research participants

Policy information about studies involving human research participants and Sex and Gender in Research.

Reporting on sex and gender	*Use the terms sex (biological attribute) and gender (shaped by social and cultural circumstances) carefully in order to avoid confusing both terms. Indicate if findings apply to only one sex or gender; describe whether sex and gender were considered in study design whether sex and/or gender was determined based on self-reporting or assigned and methods used. Provide in the source data disaggregated sex and gender data where this information has been collected, and consent has been obtained for sharing of individual-level data; provide overall numbers in this Reporting Summary. Please state if this information has not been collected. Report sex- and gender-based analyses where performed, justify reasons for lack of sex- and gender-based analysis.*
Population characteristics	*Describe the covariate-relevant population characteristics of the human research participants (e.g. age, genotypic information, past and current diagnosis and treatment categories). If you filled out the behavioural & social sciences study design questions and have nothing to add here, write "See above."*
Recruitment	*Describe how participants were recruited. Outline any potential self-selection bias or other biases that may be present and how these are likely to impact results.*
Ethics oversight	*Identify the organization(s) that approved the study protocol.*

Note that full information on the approval of the study protocol must also be provided in the manuscript.

Field-specific reporting

Please select the one below that is the best fit for your research. If you are not sure, read the appropriate sections before making your selection.

☒ Life sciences ☐ Behavioural & social sciences ☐ Ecological, evolutionary & environmental sciences

For a reference copy of the document with all sections, see nature.com/documents/nr-reporting-summary-flat.pdf

Life sciences study design

All studies must disclose on these points even when the disclosure is negative.

Sample size	The cryo-EM data collection size were determined by available holes in grid samples and allocated electron microscope beam times. The number of micrographs and protein particles used for cryo-EM structure determination are summarized in the Supplementary Figs 2, 7, 9, 12, 14, 18, 20, 23 and Supplementary Tables 1-5.<br>Interferon-beta and NF-κB assays were performed 3-4 independent times and these were enough for determine qualitative differences or significants.
Data exclusions	For cryo-EM data processing, poor-quality movie data were rejected based on ice thickness of the grids, estimated CTF resolution and total motions of the frames.
Replication	Interferon-beta and NF-κB assays were conducted at least three independent times and the replicates showed similar results.
Randomization	Random sampling was used for Half-map reconstruction. Half-map Fourier-shell correlations were used to estimate resolution of the electron density maps by applying a 0.143 threshold. Randomization were not required for interferon-beta and NF-κB assays.
Blinding	The cryo-EM particle picking, 2D classification, 3D reconstruction and validate their resolution processes were done by automatized and randomized particle selection, blinding is not relevant for cryo-EM data processing. Interferon-beta and NF-κB assays were quantitative measurement, blinding is not relevant.

Reporting for specific materials, systems and methods

We require information from authors about some types of materials, experimental systems and methods used in many studies. Here, indicate whether each material, system or method listed is relevant to your study. If you are not sure if a list item applies to your research, read the appropriate section before selecting a response.

Materials & experimental systems

n/a | Involved in the study

- [ ] | [x] Antibodies
- [ ] | [x] Eukaryotic cell lines
- [x] | [ ] Palaeontology and archaeology
- [x] | [ ] Animals and other organisms
- [x] | [ ] Clinical data
- [x] | [ ] Dual use research of concern

Methods

n/a | Involved in the study

- [x] | [ ] ChIP-seq
- [x] | [ ] Flow cytometry
- [ ] | [ ] MRI-based neuroimaging

Antibodies

Antibodies used

Commercial antibodies :
anti-FLAG antibody conjugated with horseradish peroxidase (MBL, cat no. M185-7, lot:009, clone FLA-1, 1:10,000 for western blotting), anti-β-actin antibody (Santa Cruz, sc-47778, lot: J2721, clone C4, 1:2,000 for western blotting)
Recombinantly expressed antibodies :
anti-AFLA antibody, anti-TLR3 antibody (mAb12)

Validation

(1) 1The validities of all commercial antibodies were done by manufacturer.
>> anti-FLAG antibody antibody conjugated with horseradish peroxidase recognize DDDK tag for WB, IP, flow cytometry and immunocytochemistry. The manufacturer validation informations are available at [https://www.mblbio.com/bio/g/dtl/A/?pcd=M185-3L]
>> anti-β-actin antibody recognize human, mouse, bovine β-actin for WB, IP, IF, IHC(P) and ELISA. The manufacturer validation informations are available at [https://www.scbt.com/p/beta-actin-antibody-c4]
(2) The validity of anti-TLR3 and anti-ALFA antibodies were previously confirmed by the following publication.
- Luo J, et al. Lateral clustering of TLR3:dsRNA signaling units revealed by TLR3ecd:3Fabs quaternary structure. J. Mol. Biol. 421, 112-124 (2012).
- Gotzke H, et al. The ALFA-tag is a highly versatile tool for nanobody-based bioscience applications. Nat. Commun. 10, 4403 (2019).

Eukaryotic cell lines

Policy information about cell lines and Sex and Gender in Research

Cell line source(s)

HEK293s GnTi- (ATCC, #CRL-3022), High Five (Thermo Fisher, B85502), Sf9 (Thermo Fisher, B82501), HEK-Blue Null1 (InvivoGen, hkb-null1)

Authentication

Additional authentication was not performed in this study

Mycoplasma contamination

Mycoplasma contamination test was not performed in this study

Commonly misidentified lines
(See ICLAC register)

No commonly misidentified lines were used in this study

Magnetic resonance imaging

Experimental design

Design type

Indicate task or resting state; event-related or block design.

Design specifications

Specify the number of blocks, trials or experimental units per session and/or subject, and specify the length of each trial or block (if trials are blocked) and interval between trials.

Behavioral performance measures

State number and/or type of variables recorded (e.g. correct button press, response time) and what statistics were used to establish that the subjects were performing the task as expected (e.g. mean, range, and/or standard deviation across subjects).

Acquisition

Imaging type(s)

Specify: functional, structural, diffusion, perfusion.

Field strength

Specify in Tesla

Sequence & imaging parameters

Specify the pulse sequence type (gradient echo, spin echo, etc.), imaging type (EPI, spiral, etc.), field of view, matrix size, slice thickness, orientation and TE/TR/flip angle.

Area of acquisition

State whether a whole brain scan was used OR define the area of acquisition, describing how the region was determined.

Diffusion MRI [ ] Used [x] Not used

Preprocessing

Preprocessing software	*Provide detail on software version and revision number and on specific parameters (model/functions, brain extraction, segmentation, smoothing kernel size, etc.).*
Normalization	*If data were normalized/standardized, describe the approach(es): specify linear or non-linear and define image types used for transformation OR indicate that data were not normalized and explain rationale for lack of normalization.*
Normalization template	*Describe the template used for normalization/transformation, specifying subject space or group standardized space (e.g. original Talairach, MNI305, ICBM152) OR indicate that the data were not normalized.*
Noise and artifact removal	*Describe your procedure(s) for artifact and structured noise removal, specifying motion parameters, tissue signals and physiological signals (heart rate, respiration).*
Volume censoring	*Define your software and/or method and criteria for volume censoring, and state the extent of such censoring.*

Statistical modeling & inference

Model type and settings	*Specify type (mass univariate, multivariate, RSA, predictive, etc.) and describe essential details of the model at the first and second levels (e.g. fixed, random or mixed effects; drift or auto-correlation).*
Effect(s) tested	*Define precise effect in terms of the task or stimulus conditions instead of psychological concepts and indicate whether ANOVA or factorial designs were used.*

Specify type of analysis: ☐ Whole brain ☐ ROI-based ☐ Both

Statistic type for inference<br>(See Eklund et al. 2016)	*Specify voxel-wise or cluster-wise and report all relevant parameters for cluster-wise methods.*
Correction	*Describe the type of correction and how it is obtained for multiple comparisons (e.g. FWE, FDR, permutation or Monte Carlo).*

Models & analysis

n/a	Involved in the study
☐	☐ Functional and/or effective connectivity
☐	☐ Graph analysis
☐	☐ Multivariate modeling or predictive analysis

Functional and/or effective connectivity	*Report the measures of dependence used and the model details (e.g. Pearson correlation, partial correlation, mutual information).*
Graph analysis	*Report the dependent variable and connectivity measure, specifying weighted graph or binarized graph, subject- or group-level, and the global and/or node summaries used (e.g. clustering coefficient, efficiency, etc.).*
Multivariate modeling and predictive analysis	*Specify independent variables, features extraction and dimension reduction, model, training and evaluation metrics.*
